# The impact of different forms of exercise on endothelial progenitor cells in healthy populations

**DOI:** 10.1007/s00421-022-04921-7

**Published:** 2022-03-19

**Authors:** Panagiotis Ferentinos, Costas Tsakirides, Michelle Swainson, Adam Davison, Marrissa Martyn-St James, Theocharis Ispoglou

**Affiliations:** 1 Carnegie School of Sport, Leeds Beckett University, Leeds, UK; 2 Lancaster Medical School, Faculty of Health and Medicine, Lancaster University, Lancaster, UK; 3 Flow Cytometry Facility, Leeds Institute of Cancer and Pathology, University of Leeds, St James’s University Hospital, Leeds, UK; 4 Cytec Biosciences B.V, Amsterdam, The Netherlands; 5 School of Health and Related Research, University of Sheffield, Sheffield, UK

**Keywords:** Endothelial progenitor cells, Exercise, Cardiometabolic health, Vascular health, Flow cytometry, EPC mobilisation, Resistance exercise, High intensity interval training, Moderate intensity continuous training, Aerobic training

## Abstract

Circulating endothelial progenitor cells (EPCs) contribute to vascular healing and neovascularisation, while exercise is an effective means to mobilise EPCs into the circulation. Objectives: to systematically examine the acute and chronic effects of different forms of exercise on circulating EPCs in healthy populations. Methods: Preferred Reporting Items for Systematic Reviews and Meta-analyses guidelines were followed. Results: thirty-one articles met the inclusion criteria including 747 participants aged 19 to 76 years. All included trials used flow cytometry for identification of circulating EPCs. Eight and five different EPC phenotypes were identified in the acute and chronic trials, respectively. In the acute trials, moderate intensity continuous (MICON), maximal, prolonged endurance, resistance and high intensity interval training (HIIT) exercise protocols were utilised. Prolonged endurance and resistance exercise had the most profound effect on circulating EPCs followed by maximal exercise. In the chronic trials, MICON exercise, HIIT, HIIT compared to MICON and MICON compared to exergame (exercise modality based on an interactive video game) were identified. MICON exercise had a positive effect on circulating EPCs in older sedentary individuals which was accompanied by improvements in endothelial function and arterial stiffness. Long-stage HIIT (4 min bouts) appears to be an effective means and superior than MICON exercise in mobilising circulating EPCs. In conclusion, both in acute and chronic trials the degree of exercise-induced EPC mobilisation depends upon the exercise regime applied. In future, more research is warranted to examine the dose–response relationship of different exercise forms on circulating EPCs using standardised methodology and EPC phenotype.

**Graphical abstract:**

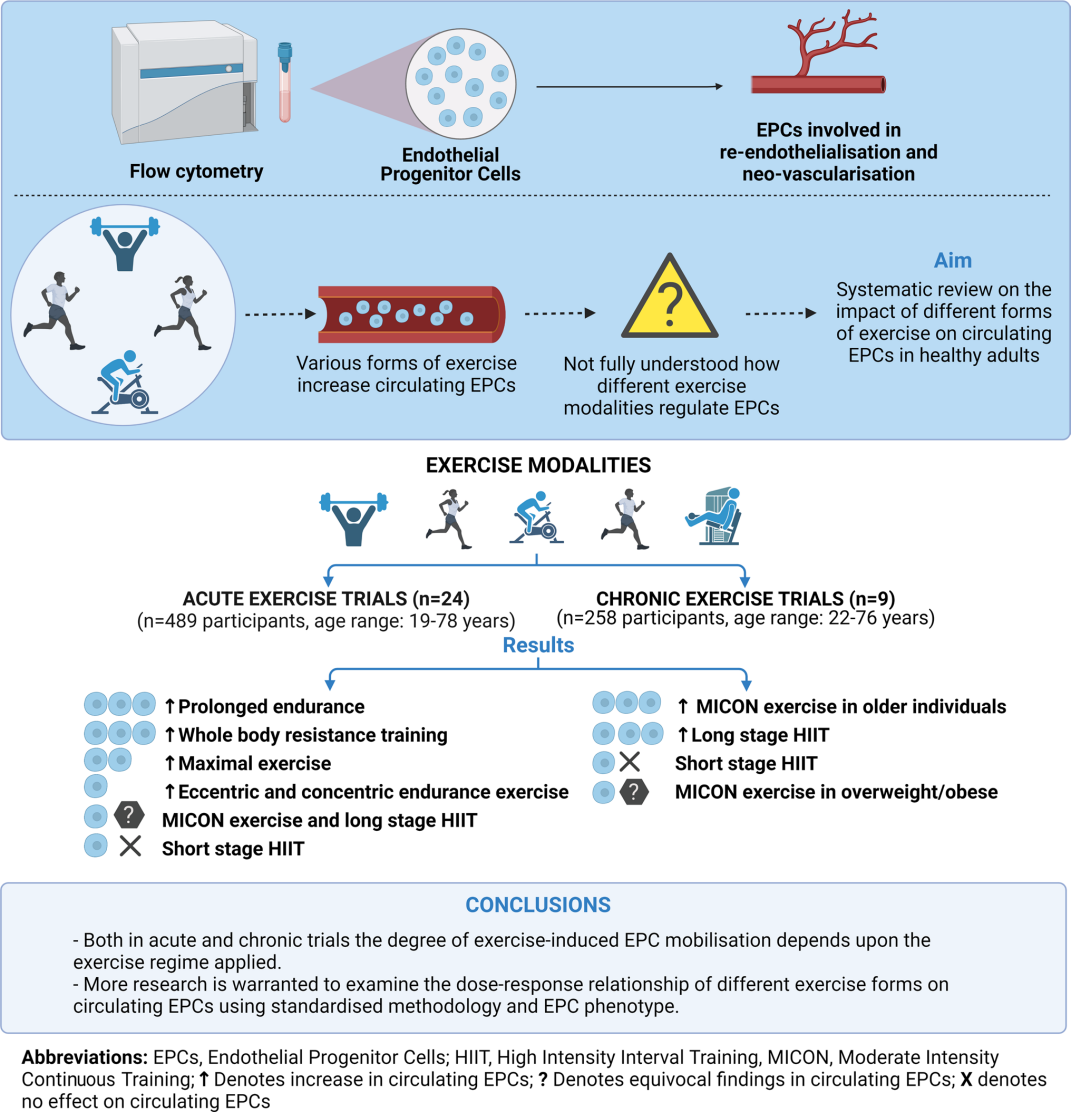

**Supplementary Information:**

The online version contains supplementary material available at 10.1007/s00421-022-04921-7.

## Introduction

The concept of vasculogenesis which was thought to occur only during embryonic development (Koutroumpi et al. 2012) was revised by Asahara et al. (1997) when they isolated CD34^+^ cells from human peripheral blood and were able to differentiate into endothelial cells. These so-called circulating endothelial progenitor cells (EPCs) consist of a heterogenous population (Hur et al. 2004) whose origin remains controversial as recent research showed that they do not originate from the bone marrow but from a niche in the vessel wall (Fujisawa et al. 2019). Although circulating EPC numbers are low, their pivotal role in re-endothelialisation and post-natal neovascularisation (Balaji et al. 2013), made them a promising tool for human tissue engineering (Plouffe et al. 2009; Tevlin et al. 2016).

Several pharmacological interventions have been implemented successfully to increase circulating EPC numbers and, consequently, improve endothelial function as assessed by flow mediated dilatation (FMD) in clinical populations including chronic heart failure, coronary artery disease and type 2 diabetes mellitus (T2DM) (Liao et al. 2010; Pelliccia et al. 2010; Erbs et al. 2011; Tousoulis et al. 2011; Oikonomou et al. 2015). However, there is evidence that healthy lifestyle modifications such as the Mediterranean diet and physical exercise can increase the population of circulating EPCs (Marin et al. 2011; De Biase et al. 2013; Guo et al. 2017; Maiorino et al. 2017) making these promising preventive strategies for the maintenance of endothelial integrity across the lifespan.

Physical exercise is a non-pharmacological tool that improves cardiorespiratory fitness, reduces inflammation, assists in the health management of people with cardiovascular risk factors, optimises muscle quantity and quality, and improves endothelial function (Goodpaster et al. 2008; Di Francescomarino et al. 2009; Gleeson et al. 2011; Vanhees et al. 2012; Lin et al. 2015). Moreover, increased physical activity levels have been shown to reduce all cause morbidity and mortality (Wei et al. 2000; Schnohr et al. 2006; Waschki et al. 2011) while low cardiorespiratory fitness is a predictor of cardiovascular events both in men and women (Kodama et al. 2009). Given the multiple beneficial effects of exercise it has been labelled as a “polypill” with similar, or even better, results compared to several pharmacological interventions (Fiuza-Luces et al. 2013). Therefore, similar to drugs, the efficacy of different exercise prescriptions (forms, intensities and durations) should be tested first by examining the acute responses in related outcomes such as peak/maximal oxygen uptake (VO_2peak/max_), FMD, and EPCs. This is particularly important regarding EPC responses since it is not currently known which is the most efficacious exercise modality for increasing circulating EPCs in healthy populations; a recent systematic review (Ferentinos et al. 2022) sheds further light regarding the efficacy of different exercise modalities however this was in populations with cardiovascular and metabolic disease. In healthy populations, previous systematic reviews and meta-analyses examined the acute effects of exercise on EPCs and found that long duration aerobic exercise had more profound results on EPCs levels compared to maximal and submaximal exercise (Silva et al. 2012) and that the numbers of EPCs remained elevated between 12-48 h post-exercise (Schmid et al. 2021). However, in their analyses they included also trials that used colony forming unit assays (CFU) for the identification of EPCs in the blood (Silva et al. 2012; Schmid et al. 2021), while the gold standard for quantification of circulating EPCs is flow cytometry (Khan et al. 2005; Fadini et al. 2012). Colony forming unit endothelial cells (CFU-ECs) are not composed of EPC progeny and are not a measure of EPCs (Hirschi et al. 2008; Fadini et al. 2012; Van Craenenbroeck et al. 2013). Notably, studies examining the chronic effects of different forms of exercise on circulating EPC numbers in healthy adults has produced varying and conflicting results possibly due to factors such as age, different exercise prescription, and presence of cardiovascular risk factors (Thijssen et al. 2006; Cesari et al. 2012; Yang et al. 2013; Niemiro et al. 2018). To date the chronic effects of different forms of exercise on circulating EPCs have not been systematically reviewed before.

Mechanistically, mobilisation and recruitment of circulating EPCs is a complex process that is mediated by various pro-angiogenic factors (Tilling et al. 2009) including chemokines [stromal cell derived factor 1 alpha (SDF-1α)], growth factors [vascular endothelial growth factor (VEGF)] and cytokines [interleukin (IL-6)], while exercise has been shown to increase them acutely and chronically alongside EPCs (Bonsignore et al. 2010; Ross et al. 2014; Tsai et al. 2016).

Therefore, the main aim of this review is to systematically summarise the current state of the literature in relation to the acute and chronic effects of different forms of exercise on the numbers of circulating EPCs, assessed only by flow cytometry in healthy non-clinical populations. A secondary aim is to provide comprehensive evidence for the responses of all the pro-angiogenic factors analysed in the trials and discuss possible underlying mechanisms of exercise-induced EPC mobilisation.

## Methods

The present systematic review was conducted in accordance with the Preferred Reporting Items for Systematic Review and Meta-analyses (PRISMA) statement (Moher et al. 2009) and was prospectively registered on PROSPERO, the international prospective register of systematic reviews. (CRD42017084552).

### Literature search

An extensive search of the relevant studies was conducted via six electronic databases (MEDLINE, Cochrane Library (CENTRAL), SPORTdiscus, CINAHL, PsycINFO and SCOPUS) from 1996 until May 2018. Two updates of the literature were subsequently conducted covering the periods between May 2018 to February 2020 and between February 2020 to April 2021 using MEDLINE database. Additionally, eligible studies were searched via relevant articles and existing reviews in the topic. The following keywords were used using Boolean operators and wild cards where appropriate: “population”, “exercise” and “endothelial progenitor cells” (Details of the strategy can be found in Supplementary Table 1 (S1)). No language restrictions applied during searching. Findings reported here relate to the healthy populations of the registered review in the PROSPERO database however the search strategy included terms for both healthy and clinical populations.

### Study selection

The types of studies included in the present systematic review were randomised control trials, non-randomised comparative trials, prospective cohort studies, controlled before–after studies and without control before–after studies. The following eligibility criteria had to be met for a study to be included: (1) the subjects were independently living individuals of good health, (2) the study used various forms of structured exercise programmes or various forms of acute exercise bouts and (3) studies that used flow cytometry as their primary method to quantify circulating EPCs. Regarding the definition of EPC phenotype, despite that there is not a unique antibody combination for EPC enumeration from flow cytometry, we followed Fadini’s et al. recommendation that the phenotype should have at least one marker of immaturity/stemness (e.g. CD34, CD133) and at least one marker that represents endothelial lineage (e.g. KDR, CD31) (Fadini et al. 2008a). We excluded animal studies, studies which included participants under 18 years old or pregnant women and studies that included a dietary intervention. All citations along with abstracts were extracted and imported to EndNote version X9 and duplicates were removed. The titles and abstracts were scanned and assessed independently by two reviewers (PF and MS). Studies that deemed to meet the inclusion criteria were included in the present systematic review.

### Data extraction and quality assessment

After reviewing the full paper of all eligible studies, the data were extracted using a standardised extraction sheet in Microsoft excel (Office 365 Plus) by three independent reviewers (PF, CT and MS) and included: (1) Study information (Author, year); (2) Study population (clinical condition, age, sex, fitness status); (3) Exercise intervention (Acute; defined as a single bout of physical activity (Sellami et al. 2018) /Chronic; defined as repeated number of bouts of physical activity during short or long-term period of time (Sellami et al. 2018); (4) Exercise protocol (type of exercise, intensity, duration); (5) Primary outcomes (EPC phenotype, unit of measure, blood sampling time); (6) Secondary outcomes (cytokines, growth factors, chemokines, FMD, maximal oxygen uptake (VO_2max_)). After data extraction, a meeting was held by the three reviewers to cross-check the extracted data. Any disagreements were resolved by discussion. Data not provided in the text or tables were extracted from figures using a semi-automated graph digitizer software (WebPlotDigitizer).

Study quality was evaluated through consensus by PF and TI, using different quality assessment tools as appropriate for each study design. Randomised controlled trials and non-randomised controlled trials were assessed using TESTEX; the tool for the assessment of study quality and reporting in exercise is a 15 point scale that assesses study quality criteria (maximum five points) and study reporting criteria (maximum 10 points) (Smart et al. 2015). Prospective cohort, controlled before-after and without control before- after studies were assessed with the appropriate study quality assessment tools from the National Heart, Lung and Blood Institute (NHLBI) (National Heart). The quality assessment tools from NHBLI include a series of questions focussing on the critical appraisal of the internal validity of a study. The potential responses for each question are “yes”, “no”, or “cannot determine/not reported /not applicable”. The final rating for each study can be “good”, “fair” or “poor” quality.

### Evidence synthesis

Due to the lack of trials with a common design (e.g. blood sampling time) that would allow us to perform a meta-analysis for assessing the effects of different exercise modalities on circulating EPCs, and because of the variety of methodological assessment of EPCs (e.g. variation of EPC phenotypes), we opted to undertake a narrative synthesis using the Synthesis Without Meta-Analysis in systematic reviews: reporting guideline (Campbell et al. 2020). The narrative synthesis was based on constructing evidence tables of extracted study data mirrored by a narrative synthesis across studies. The results from the studies presented in two main categories: studies that examined acute effects and studies that examined chronic effects. Tables reporting the numbers of blood collection points and fasting/non-fasting status were arranged alphabetically. Quality assessment results arranged based on the quality score from the highest to the lowest. The graphical abstract and Fig. 2 were created with BioRender.com.

## Results

**Fig. 1:**
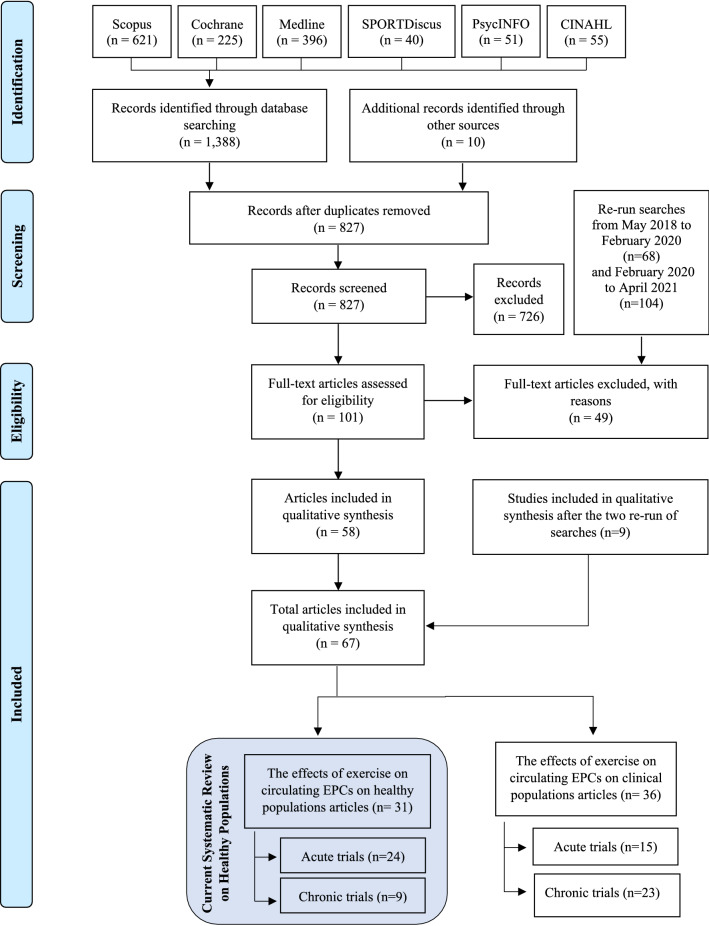
PRISMA flow diagram illustrating the searching strategy and selection of the articles used in this systematic review

From the electronic search 1,388 articles were identified. In addition, the manual search from previous reviews identified another 10 yielding in total 1,398 articles. After removal of duplicates, 827 articles were reviewed based on title and abstract and, after the first sifting, 101 potential eligible articles remained for full-text screening. A total of 58 articles met the inclusion criteria for this review. After the re-run of searches, there were nine additional articles eligible for this review therefore, the total number of articles included in the systematic review was 67 as shown in the PRISMA flow diagram (Fig. 1). Of the 67 eligible articles, 31 are included in the present systematic review which focuses on healthy populations. The remaining 36 articles formed the basis of a systematic review focussing on populations with cardiovascular and metabolic disease (Ferentinos et al. 2022). A full list of 49 articles, accompanied by reason, excluded for the review can be found in Supplementary Table S2 (S2).

### Overview of the study characteristics

Thirty-one articles yielded 33 trials in total. One article (Thijssen et al. 2006) included one acute and one chronic exercise trial and another article (Bonsignore et al. 2010) included two acute exercise trials. Overall, the trials included 747 apparently healthy individuals (82% males and 18% females) with ages ranging between 19 to 76 years.

### Acute trial characteristics and intervention details

There were 24 trials that investigated the acute effects of exercise on circulating EPCs, with 489 total participants (Table 1). Interestingly, 18 (75%) of the acute trials included solely male participants (Thijssen et al. 2006; Adams et al. 2008; Bonsignore et al. 2010; Chang et al. 2015; Cubbon et al. 2010; Kruger et al. 2016, 2015; Laufs et al. 2005; Lockard et al. 2010; Mobius-Winkler et al. 2009; Niemiro et al. 2017; Ross et al. 2018, 2014; Stromberg et al. 2017; Yang et al. 2007; Montgomery et al. 2019; Sapp et al. 2019), two trials included only female participants (Ribeiro et al. 2017; Harris et al. 2017), two included two separate groups based on sex (Shill et al. 2016; Anz et al. 2019) and two used a mixed sample (Van Craenenbroeck et al. 2008b; O'Carroll et al. 2019). The age range of the participants was from 19 to 76 years with 60.9% of the participants < 35 years old.

**Table 1:** Summary of trials examining the acute effects of different exercise modalities on EPCs in healthy populations

Study	Study design	Participant characteristics	Exercise Prescription	EPC phenotype identified by flow cytometry and units in brackets	Results on circulating EPCs and other major findings
**Trials that included MICON exercise**					
Chang et al. (2015)	Single arm	**EX group**: n = 5 healthy, 100%males, 29.8 years (26–36)	**Modality:** Aerobic exercise on treadmill. **Duration:** 30 min. **Intensity:** HR > 140 bpm	KDR^+^/CD11b^−^/ CD34^+^/AC133^+^ (% PBMNCs)	** ↔ 10 min post but 3.6-fold ↑ 24 h post** Pre (0.10 ± 0.02) vs 30 min Post (0.11 ± 0.04), *P* > 0.05. Pre (0.10 ± 0.02) vs 24 h Post (0.36 ± 0.09), *P* < 0.05. ↑ SDF-1α (10 min post);↑ EPO (24 h post)
Cubbon et al. (2010)	Independent groups, before and after	**EX group 1** (White European): n = 15 healthy, 100% males, 28.2 ± 1.3yrs,VO_2max_: 48.2 ± 2.7 ml.kg^−1^.min^−1^ **EX group 2** (South Asian): n = 15 healthy, 100% males, 30.0 ± 1.3yrs. VO_2max_: 38.0 ± 1.8 ml.kg^−1^.min^−1^	**Modality:** Aerobic exercise on cycle ergometer. **Duration:** 30 min. **Intensity:** 80% of individual lactate threshold	CD34^+^/KDR^+^ and CD133^+^/CD34^+^/KDR^+^ (% Lymphocytes)	**Reduced EPC mobilisation after exercise in EX group 2 (South Asian group) vs EX group 1 (White European)** CD34^+^/KDR^+^: 53.2% ± 6.9% vs 85.4% ± 5.1%, *P* = 0.001.- CD133^+^/CD34^+^/KDR^+^: 48.3% ± 8.8% vs 78.4 ± 5.7%, *P* = 0.05 Relationship between baseline FMD with CD34^+^/KDR^+^ EPCs as an entire study sample (r = 0.41, *P* = 0.023) and CD133^+^/CD34^+^/KDR^+^ EPCs (r = 0.39, *P* = 0.035)
Lansford et al. (2016)	Independent groups, before and after	**EX group 1:** n = 16 healthy, 100% males, 24.5 ± 0.8yrs, VO_2peak_: 49.2 ± 1.4 ml.kg^−1^.min^−1^ **EX group 2:** n = 10 healthy, 100%females, 22.40 ± 0.52yrs, VO_2peak_: 44.4 ± 2.0 ml.kg^−1^.min^−1^	**Modality:** Aerobic exercise on cycle ergometer. **Duration:** **EX group 1:** 43.6 ± 1.5 min **EX group 2:** 62.7 ± 1.9 min. **Intensity:** 60–70%VO_2max_	CD34^+^/KDR^+^ (PBMNCs/50,000 events)	** ↔ Post in any of the groups** **EX group 1:**Pre 190.6 ± 24.9 vs Post 389.5 ± 139.2, *P* > 0.05. **EX group 2**:Pre 198.9 ± 34.8 vs Post 184.0 ± 43, *P* > 0.05
Laufs et al. (2005)	Randomised cross over	**EX group:** n = 25 healthy, 100% males, 28.4 ± 6.5yrs, VO_2max_: 57.8 ± 2.7 ml.kg^−1^.min^−1^	**Modality 1:** Intensive running on 400 m track. **Duration:**30 min **Intensity:** 100% velocity of the IAT **Modality 2:** Moderate running on 400 m track. **Duration:** 30 min. **Intensity:** 80% velocity of the IAT **Modality 3:** Moderate short duration running on 400 m track. **Duration:** 10 min. **Intensity:** 80% velocity of the IAT	CD34^+^/KDR^+^ (Cells/10^5^ events events)	**↑ of EPCs at 10 min Post in intensive (Modality 1) and moderate 30 min running (Modality 2) but not in short term 10 min run (Modality 3)** **Modality 1:** Pre 21.0 ± 9.5 vs Post 43.3 ± 17.4, *P* < 0.01 **Modality 2:** Pre 24.5 ± 10.3 vs Post 64.4 ± 21.2, *P* < 0.01 **Modality 3:** Pre 26.0 ± 12.2 vs Post 27.5 ± 11.4, *P* > 0.05 ↔ VEGF; ↔ Cortisol
Lockard et al. (2010)	Independent groups, before and after	**EX group 1:** n = 12 healthy highly active, 100% males, 62.0 ± 1.6yrs, VO_2max_: 50.0 ± 1.3 ml.kg^−1^.min^−1^ **EX group 2:** n = 11 healthy less active, 100% males, 65.0 ± 1.5yrs, VO_2max_: 28.2 ± 1.8 ml.kg^−1^.min^−1^	**Modality:** Aerobic exercise on treadmill. **Duration:** 30 min. **Intensity:** 75 ± 5%VO_max_	CD34^+^/KDR^+^ (Cells/10^5^events)	** ↔ **Post in any of the groups **EX group 1:**Pre 84.0 ± 22.9 vs Post 132.2 ± 59.7, *P* = 0.326. **EX group 2:** Pre 41.3 ± 23.9 vs Post 80.1 ± 84.4, *P* = 0.471
Niemiro et al. (2017)	Single arm	**EX group:** n = 7 healthy, 100% males, 25.3 ± 2.4yrs, VO_2max_: 60.9 ± 2.74 ml.kg^−1^.min^−1^	**Modality:** Aerobic exercise on treadmill. **Duration:** 60 min. **Intensity:** 70%VO_peak_	CD45^−^/CD34^+^/CD31^+^ (Cells/mL)	** ↔ during or after exercise at any time point (*P* > 0.05)** ↑ plasma SDF-1α at 40 and 60 min during exercise and 15 min Post ↑ plasma SCF at 40 and 60 min during exercise and 15 and 60 Post
Ross et al. (2018)	Independent groups, before and after	**EX group 1:** n = 8 healthy young, 100% males, 23 ± 2yrs, VO_2max_: 48.8 ± 8.2 ml.kg^−1^.min^−1^ **EX group 2:** n = 9 healthy old, 100% males, 65 ± 3yrs, VO_2max_: 35.1 ± 6.7 ml.kg^−1^.min^−1^	**Modality:** Aerobic exercise on cycle ergometer. **Duration:** 30 min. **Intensity:** 70%VO_max_	CD34^+^/ KDR^+^/CD45^dim^ Cells/mL	**↑ after exercise in both groups (*P* = 0.003). Absolute EPC mobilisation between EX group 1 vs EX group 2 (212 ± 72 vs 67 ± 23, *P* = 0.076)** ↑ VEGF in both groups; ↑ Cortisol in both groups with significant exercise x age interaction; ↔ SDF-1α and G-CSF (in both groups)
Strömberg et al. (2017)	Single arm	**EX group:** n = 10 healthy, 100% males, 25(20,31) yrs,VO_2max_: 54(43,59) ml.kg^−1^.min^−1^	**Modality:** Aerobic exercise on a cycle ergometer. **Duration:** 60 min **Intensity:** 50%VO_2max_ in 20 first min and 65%VO_2max_ for the next 40 min	CD34^+^/ KDR^+^/CD45^dim^ Cells/mL	**A trend to ↑ Post EX (*P* = 0.08)** Pre 0.3 ± 0.2 vs Post 1.0 ± 1.2, *P* > 0.05. Pre 0.3 ± 0.2 vs 30 min Post1.2 ± 2.4, *P* > 0.05. Pre 0.3 ± 0.2 vs 2 h Post, 1.8 ± 2.2, *P* > 0.05. ↔ G-CSF; ↔ SDF-1α; ↔ VEGF
**Trials that included maximal exercise**					
Bonsignore et al. (2010)	Single arm	**EX group:** n = 8 healthy, 100% males, 43.4 ± 10.9yrs	**Modality:** 1500 m field test. **Duration:** 5 min 35 s ± 35 s. **Intensity:** 101 ± 5% predicted HRmax	CD34^+^/KDR^+^ (Cells/mL)	** ~ three-fold increase after the 1500 m field test** Pre 0.21 ± 0.14 vs Post 0.55 ± 0.39, *P* < 0.01. ↑ IL-6;↑ HGF; ↔ VEGF-A; ↑ VEGF-C; ↔ VEGF-D; ↔ Ang-1; ↑ Ang-2; ↔ Ang-1/Ang-2; ↔ CK; ↑ SCF
Shill et al. (2016)	Independent groups, before and after	**EX group 1:** n = 11 healthy, 100% males, 24.8 ± 1.6yrs,VO_2max_: 50.7 ± 1.4 ml.kg^−1^.min^−1^ **EX group 2:** n = 11 healthy, 100% females, 23.7 ± 1.6yrs,VO_2max_: 42.6 ± 1.9 ml.kg^−1^.min^−1^	**Modality:** VO_2max_ test on a treadmill. **Duration:** 6-12 min. **Intensity:** Maximal	CD34^+^/KDR^+^ (PBMNCs/100,000 events)	**23% ↑ in EX group 1 (males)** **46% ↑ in EX group 2 (females)** **33% ↑ combined** **EX group 1:** Pre 4546.6 ± 652.7 vs 5584.9 ± 806.3, *P* < 0.05. **EX group 2:** Pre 3443.9 ± 377.9 vs 5044.0 ± 318.7, *P* < 0.001. **Combined:** Pre 3995.3 ± 387.2 vs 5314.5 ± 427.1, *P* < 0.001 No relationship between VO_2max_ and exercise-induced EPCs
Thijssen et al. (2006)	Independent groups, before and after	**EX group 1:** n = 8 healthy sedentary young, 100% males, 19-28yrs,VO_2max_: 49.0 ± 4.0 ml.kg^−1^.min^−1^ **EX group 2:** n = 8 healthy endurance, 100% males, 18-28yrs, VO_2max_: 58.5 ± 7.5 ml.kg^−1^.min^−1^ **EX group 3:** n = 8 healthy sedentary old, 100% males, 67-76yrs, VO_2max_: 30.8 ± 4.8 ml.kg^−1^.min^−1^	**Modality:** VO_2max_ test on a treadmill. **Intensity:** Maximal	CD34^+^/ KDR^+^ (Cells/mL)	** ↔ after exercise in any group** **EX group 1:** Pre 154 ± 43 vs Post 199 ± 73, *P* > 0.05. **EX group 2:** Pre 185 ± 96 vs Post 367 ± 99,* P* > 0.05. **EX group 3:** Pre 35 ± 12 vs Post 32 ± 34,* P* > 0.05 ↑ VEGF (only in EX group 1); relationship between baseline EPCs with exercise-induced EPCs (combined groups) (r = 0.47, *P* = 0.007); no relationship between exercise-induced EPCs and VEGF in young (r^2^ = 0.001, *P* = 0.93) and in older men (r^2^ = 0.04, *P* = 0.63); no relationship between VO_2max_ with baseline EPCs (r^2^ = 0.10, *P* = 0.23) and exercise-induced EPCs (r^2^ = 0.20, *P* = 0.08) in young men; no relationship between VO_2max_ with baseline EPCs (r^2^ = 0.05, *P* = 0.42) and exercise-induced EPCs (r^2^ = 0.07, *P* = 0.51) in older men
Van Craenenbroeck et al. (2008a, b)	Independent groups, before and after	**EX group 1:** n = 11 healthy, 55% males, 23.9 ± 1.4yrs, VO_2peak_: 50.6 ± 10.3 ml.kg^−1^.min^−1^ **EX group 2:** n = 14 healthy, 64% males, 36.2 ± 9.3yrs VO_2peak_: 46.0 ± 11.8 ml.kg^−1^.min^−1^	**Modality:** CPET on cycle ergometer. **Intensity:** Maximal	CD34^+^/KDR^+^ (Cells/mL)	**↑ by 76% and 69% Post in group 1 and group 2 respectively** **EX group 1:** Pre 15.4 ± 10.7 vs Post 27.2 ± 13.7, *P* = 0.01**. EX group 2:** Pre 30.9 ± 14.6 vs Post 52.5 ± 42.6, *P* = 0.03 ↔ VEGF; ↔ NOx Relationship between % increase EPCs with LDL (EX group 1: r = 0.745, *P* = 0.008; EX group 2: r = 0.569, *P* = 0.034) and TC/HDL (EX group 1: r = 0.717, *P* = 0.013; EX group 2: r = 0.047, *P* = 0.538); EX group 1 reverse relationship between % increase EPCs with VO_2peak_ (r = 0.636, *P* = 0.035 and VO_2_ at AT; r = -0.83, *P* = 0.003); no relationship between VEGF increase with EPCs (*P* > 0.05)
Yang et al. (2007)	Single arm	**EX group:** n = 16 healthy, 100% males, 25.1 ± 2.7yrs	**Modality:** modified Bruce treadmill protocol (5.5 km.h^−1^, 14% grade). **Intensity:** Maximal. **Duration:** 9.6 ± 2.2 min	CD34^+^/KDR^+^ (% PBMNCs)	**↑ Pre 0.030 ± 0.012 vs Post 0.052 ± 0.022, *P* < 0.05** ↑ NOx; ↔ EGF; ↔ GM-CSF; correlation between increase in plasma NOx with increase in CD34^+^/KDR^+^ EPCs (r = 0.70, *P* < 0.05)
**Trials that included prolonged endurance exercise**					
Adams et al., (2008)	Single arm	**EX group:** n = 68 healthy, 100% males, 57 ± 6yrs	**Modality:** Marathon race. **Duration:** 4 h,11 min ± 4 min. Intensity: N/A	CD34^+^/KDR^+^ (Cells/mL)	** ↔ between pre and post: Pre 117 ± 8 vs Post 128 ± 9, *P* = 0.33** ↓ Plasma VEGF; no relationship between EPCs with VEGF
Bonsignore et al. (2010)	Single arm	**EX group:** n = 9 healthy, 100% males, 43.6 ± 11.6yrs	**Modality:** Marathon race. **Duration:** 191 ± 26 min **Intensity:** N/A	CD34^+^/KDR^+^ (Cells/mL)	** ~ two-fold increase after Marathon race** Pre 0.23 ± 0.14 vs post-race 0.44 ± 0.18, *P* < 0.005.Pre 0.23 ± 0.14 vs ~ 18 h Post 0.23 ± 0.1, *P* > 0.05. ↑ IL-6; ↑ HGF; ↔ VEGF-A; ↑ VEGF-C; ↔ VEGF-D; ↑ Ang-1; ↑ Ang-2; ↔ Ang-1/Ang-2; ↑ CK; ↑ SCF
Mobius-Winkler et al. (2009)	Single arm	**EX group:** n = 18 healthy, 100% males, 32.4 ± 2.3yrs, VO_2max_: 59.8 ± 2.3 ml.kg^−1^.min^−1^	**Modality:** Aerobic exercise on cycle ergometer. **Duration:** 240 min. **Intensity:** 70% of the IAT	CD34^+^/KDR^+^ and CD133^+^/KDR^+^ (Cells/mL)	**CD34**^**+**^**/KDR**^**+**^** had a peak of 5.5-fold ↑ at 240 min of exercise. CD133**^**+**^**/KDR**^**+**^** had a peak of 3.5-fold ↑ at 210 min of exercise.** CD34^+^/KDR^+^: Pre 35.3 ± 6.2 vs 210 min EX 146.0 ± 24.9, *P* < 0.05. Pre 35.3 ± 6.2 vs 240 min EX 155.7 ± 22.2, *P* < 0.05. CD133^+^/KDR^+^: Pre 86.0 ± 12.0 vs 210 min EX 275.9 ± 43.0, *P* < 0.001. Pre 86.0 ± 12.0 vs 240 min EX 264.0 ± 37.0, *P* < 0.001. Pre 86.0 ± 12.0 vs 30 min Post 256.8 ± 37.1, *P* < 0.01. Pre 86.0 ± 12.0 vs 60 min Post 228.2 ± 27.4, *P* < 0.05. Pre 86.0 ± 12.0 vs 120 min Post 274.7 ± 33.8, *P* < 0.01 ↑ 1.9-fold VEGF at 10 min Post; ↑ 16.5-fold IL-6 at 30 min Post; relationship between ΔCD133^+^/KDR^+^ EPCs and ΔVEGF (r = 0.67, *P* = 0.0045); no relationship between ΔCD34^+^/KDR^+^ EPCs and ΔVEGF (r = 0.045, *P* = 0.86)
**Trials that included resistance exercise**					
Kruger et al. (2015)	Independent groups, before and after	**EX group 1** (Concentric endurance): n = 12 healthy, 100% males, 26.4 ± 1.3yrs, VO_2max_: 45.8 ± 4.2 ml.kg^−1^.min^−1^ **EX group 2** (Resistance exercise): n = 12 healthy, 100% males, 25.9 ± 4.7yrs. **EX group 3** (Eccentric endurance): n = 12 healthy, 100% males, 25.5 ± 4.3yrs, VO_2max_: 46.7 ± 3.9 ml.kg^−1^.min^−1^	**EX group 1.** Modality: Concentric endurance cycling. Duration: 43 ± 5 min. Intensity: 80%VO_2max_ **EX group 2.** Modality: Resistance exercise including bench press, latissimus pull down, seated rows, shoulder press, bicep curls and leg curls. Duration: 90 ± 5 min. Intensity: 75%1RM **EX group 3.**Modality: Eccentric downhill treadmill running. Duration: 51 ± 6 min.Intensity: 80% VO_2max_	CD34^+^/KDR^+^/CD45^−^ (Cells/mL)	**Group 1 ↑ at 3 h Post. Group 2 ↑ at 3 h and 24 h Post. Group 3 ↑ immediate post, at 3 h, 24 h and 48 h Post** **Group 1:** Pre 80.1 ± 30.9 vs Post 139.7 ± 30.8, *P* > 0.05. Pre 80.1 ± 30.9 vs 3 h Post 160.3 ± 43.1, *P* < 0.05. Pre 80.1 ± 30.9 vs 24 h Post 92.5 ± , *P* > 0.05. **Group 2:** Pre 84.2 ± 24.7 vs Post 170.5 ± 47.3, *P* > 0.05. Pre 84.2 ± 24.7 vs 3 h Post 250.7 ± 47.2, *P* < 0.05. Pre 84.2 ± 24.7 vs 24 h Post 425.3 ± 113.1, *P* < 0.05. Pre 84.2 ± 24.7 vs 48 h Post 113.0 ± 49.3, *P* > 0.05. **Group 3:** Pre 106.8 ± 45.2 vs Post 297.9 ± 78.1, *P* < 0.05. Pre 106.8 ± 45.2 vs 3 h Post 256.8 ± 330.8, *P* < 0.05. Pre 106.8 ± 45.2 vs 24 h Post 256.0 ± 96, *P* < 0.05. Pre 106.8 ± 45.2 vs 48 h Post 228.1 ± 322.6, *P* < 0.05 ↑ CK and G-CSF (in all groups Post); ↑ CRP (in EX groups 2 and 3 Post); relationship between G-CSF and EPCs at 3 h Post (r = 0.54, *P* < 0.05)
Ribeiro et al. (2017)	Independent groups, before and after	**EX group 1:** n = 13 healthy, 100% females, 20.7 ± 1.7yrs **EX group 2:** n = 12 healthy, 100% females, 21.0 ± 0.9yrs **EX group 3:** n = 13 healthy, 100% females, 20.9 ± 1.4yrs	**Modality:** Resistance exercise (3 × 12 repetitions including barbell bench press standing barbell curl, dumbbell squat and standing dumbbell upright row). **Duration:** ~ 30 min **Intensity:** **EX group 1:** 60%1RM **EX group 2:** 70%1RM **EX group 3:** 80%1RM	CD34^+^/ KDR^+^/ CD45^dim^ (% Leucocytes)	**↑ in all protocols Post with highest increase in EX group 3 (80% 1RM). EPCs reached peak levels at 6 h Post at EX group 3 (80%1RM) and ~ 5 min Post for EX group 1 and EX group 2 (60% and 70%1RM respectively)** **EX group 1:** Pre 0.00869 ± 0.00097 vs Post 0.01075, *P* = 0.038. **EX group 2:** Pre 0.00765 ± 0.00042 vs Post 0.01095 ± 0.00081, *P* < 0.001. Pre 0.00765 ± 0.00042 vs 6 h Post 0.00938 ± 0.00054, *P* = 0.001. Pre 0.00765 ± 0.00042 vs 24 h Post 0.00679 ± 0.00044, *P* = 0.029 **EX group 3:**Pre 0.00787 ± 0.00037 vs Post 0.01158 ± 0.00093, *P* < 0.001. Pre 0.00787 ± 0.00037 vs 6 h Post 0.01397 ± 0.00093, *P* < 0.001 ↑ VEGF at 5 min,6 h and 24 h Post at 70% and 80%1RM and at 5 min Post only at 60%1RM; ↑ HIF-1α at 5 min,6 h and 24 h Post at 80%1RM; ↑ HIF-1α at 6 h Post at 70%1RM and no change in 60%1RM; ↑ EPO at all-time points in all groups apart from 60%1RM which peaked at 5 min Post only; ↔ SDF-1α (in all groups); relationship between change in EPCs with VEGF (r = 0.492, *P* = 0.002) and HIF-1α (r = 0.388, *P* = 0.016) from baseline to 6 h Post
Ross et al.(2014)	Single arm	**EX group:** n = 13 healthy, 100% males, 22.4 ± 0.5yrs	**Modality:** Resistance exercise (3 circuits × 15 repetitions including leg press, seated chest press, leg curl, Lat pulldown, knee extension, triceps pushdown) with 1 min rest between each circuit **Duration:** 12.1 ± 0.6 min **Intensity:** 15RM	CD34^+^/ KDR^+^/CD45^dim^ (Cells/mL)	**↑ 2 h Post** Pre 88.7 ± 28.0 vs 10 min Post 100.0 ± 20.0, *P* > 0.05. Pre 88.7 ± 28.0 vs 2 h Post 137.0 ± 26.3, *P* < 0.01. Pre 88.7 ± 28.0 vs 24 h Post 134.7 ± 29.3, *P* > 0.05.↑ VEGF-A, VEGF-C at 10 min Post; ↑ VEGF-D at 10 min and 2 h Post; ↑ G-CSF at 2 h Post; ↑ MMP-2 at 10 min and 2 h Post; ↑ MMP-9 at 10 min and 2 h Post
Montgomery et al. (2019)	Single arm	**EX group:** n = 9 healthy, 100% males, 21 ± 1yrs	**Modality:** Unilateral knee extension on an isokinetic dynamometer (4sets in total including 1 × 30reps and 3 × 15reps with 30 s recovery)	CD34^+^/KDR^+^ and CD34^+^/CD45^dim^/KDR^+^ (Cells/mL)	**↑ in both phenotypes** CD34^+^/KDR^+^: Pre 269 ± 42 vs Post 573.0 ± 90, *P* < 0.008. Pre 269 ± 42 vs 30 min Post 564 ± 128, *P* < 0.010. CD34^+^/CD45^dim^/KDR^+^: Pre 129 ± 21 vs Post 255 ± 46, *P* > 0.05. Pre 129 ± 21 vs 30 min Post 313 ± 103, *P* < 0.010
**Trials that compared HIIT to MICON**					
O’Carroll et al. (2019)	Crossover	**EX group:** n = 12 healthy active, 67% males, 29 ± 2yrs, VO_2peak_:44.3 ± 1.8 ml.kg^−1^.min^−1^	**Modality 1:** Aerobic exercise on a cycle ergometer. **Duration:** 45 min. **Intensity:** 70% VO_2peak_ **Modality 2:** Interval exercise 6 × 20 s sprints with 2 min low active or passive recovery. **Duration:** 12 min. **Intensity:** Maximum	CD34^+^/CD45^dim^/KDR^+^ (Cells/mL)	**No trial by time point interaction** **(*P* = 0.88). Main effect for timepoint with EPCs increased Post and returns to baseline after 2 h post Ex (*P* < 0.05). Modality 1:** Pre 245 ± 55 vs Post 331 ± 83, *P* > 0.05. Pre 245 ± 55 vs 2 h Post 267 ± 65,* P* > 0.05.Pre 245 ± 55 vs 24 h Post 231 ± 34,* P* > 0.05. **Modality 2:** Pre 193 ± 37 vs Post 260 ± 35, *P* > 0.05. Pre 193 ± 37 vs 2 h Post 193 ± 36, *P* > 0.05. Pre 193 ± 37 vs 24 h Post 114 ± 23, *P* > 0.05
Harris et al. (2017)	Randomised crossover	**EX group**: n = 15 healthy post-menopausal, 100% females, 63 ± 4yrs,VO_2peak_: 44.3 ± 1.8 ml.kg^−1^.min^−1^	**Modality 1:** Aerobic exercise on a cycle ergometer. **Duration:** 30 min. Intensity: 80% work rate at LT. **Modality 2:** Interval exercise (10:20 s, work: recovery). **Duration:** 30 min. **Intensity:** 90%work rate at VO_2peak_ **Modality 3:** Interval exercise on a cycle ergometer (30:60 s, work: recovery). **Duration:** 30 min. **Intensity:** 90% work rate at LT	CD34^+^/ KDR^+^ and CD34^+^/KDR^+^/CD133^+^ (Cells/mL)	**No time by exercise interaction (*P* > 0.05). Modality 1:** CD34^+^/ KDR^+^: Pre 251 ± 176 vs 30 min Post 108 ± 119, *P* > 0.05. CD34^+^/KDR^+^/CD133^+^: Pre 68 ± 102 vs 30 min Post 11 ± 13, *P* > 0.05 **Modality 2:** CD34^+^/ KDR^+^: Pre 251 ± 176 vs 30 min Post 244 ± 188, *P* > 0.05 CD34^+^/KDR^+^/CD133^+^:Pre 68 ± 102 vs 30 min Post 40 ± 85, *P* > 0.05 **Modality 3:** CD34^+^/ KDR^+^: Pre 251 ± 176 vs 30 min Post 144 ± 201, *P* > 0.05. CD34^+^/KDR^+^/CD133^+^: Pre 68 ± 102 vs 30 min Post 14 ± 26, *P* > 0.05. ↔ FMD
Sapp et al. (2019)	Randomised crossover	**EX group:** n = 10 healthy moderately active, 100% males, 22 ± 2yrs	**Modality 1:** Aerobic exercise on a cycle ergometer. **Duration:** 30 min. **Intensity:** 60% PPO **Modality 2:** Interval exercise (3:4 min, work: recovery). Duration: 30 min. Intensity: 85%PPO:40%PPO	CD34^+^/ CD31^+^/ CD45^dim/−^ (Cells/500,000 events)	**↓ 34% and 21% following modality 1 and 2 respectively** **Modality 1:** Pre 145 ± 18 vs Post 94 ± 19, *P* = 0.02 **Modality 2:** Pre 139 ± 16 vs Post 110 ± 14, *P* < 0.001 ↔ FMD
Kruger et al. (2016)	Randomised crossover	**EX group:** n = 23 healthy, 100% males, 25.7 ± 3.2yrs, VO_2max_: 45.33 ± 5.41 ml.kg^1^.min^1^	**Modality 1:** Aerobic exercise on cycle ergometer. **Duration:** 30 min; **Intensity:** 70%VO_2max_ **Modality 2:** HIIT on cycle ergometer (5 sets x 3 min at 90%PPO with 3 min unloaded active recovery). **Duration:** 27 min	CD34^+^/KDR^+^/CD45^−^ (Cells/ul)	**↑ Post in both protocols without any significant difference between them. Modality 1:** Pre 1.82 ± 0.28 vs Post 4.16 ± 0.59, *P* = 0.035. Pre 1.82 ± 0.28 vs 3 h Post 3.06 ± 0.35, *P* > 0.05. Pre 1.82 ± 0.28 vs 24 h Post 3.36 ± 0.69, *P* > 0.05. **Modality 2:** Pre 2.47 ± 0.34 vs Post 3.21 ± 0.32, *P* = 0.027. Pre 2.47 ± 0.34 vs 3 h Post 2.96 ± 0.30, *P* > 0.05. Pre 2.47 ± 0.34 vs 24 h Post 2.86 ± 0.30, *P* > 0.05

*Ang -1/2* (angiopoietin 1/2), *Ang-1/Ang-2 (*ratio between angiopoietin 1 and angiopoietin 2), *AT* (anaerobic threshold), *CK* (creatine kinase), *CPET* (cardiopulmonary exercise test), *EPCs* (endothelial progenitor cells), *EPO* (erythropoietin), *EX* (exercise), *FMD* (flow mediated dilatation), *G-CSF (*granulocyte colony stimulating factor), *GM-CSF (*granulocyte macrophage colony stimulating factor), *HIIT (*high intensity interval training), *HIF-1α (*hypoxic inducible factor 1 alpha), *HGH* (hepatocyte growth factor), *HR* (heart rate), *HRmax* (maximum heart rate), *IAT* (individual anaerobic threshold), *IL-6* (interleukin 6), *LDL* (low density lipoprotein cholesterol), *LT (*lactate threshold), min (minutes), *MJ* (mega joules), *MICON* (moderate intensity continuous training), *mL* (millilitres), *MMP-2/9* (matrix metalloproteinase 2/9), *N/A* (not applied), *NOx (*nitric oxide metabolites (nitrite/nitrate)), *PBMNCs* (peripheral blood mononuclear cells), *PPO* (peak power output), *SCF* (stem cell factor), *SDF-1α (*stromal cell derived factor 1 alpha), *VEGF* (vascular endothelial growth factor), *VO*_*2peak/max*_* (*peak/max oxygen uptake), *yrs* (years), *μL* (microliter), *1RM* (one repetition maximum strength), ↑ (indicates significant increase), ↓ (indicates significant decrease), ↔ (indicates no significant change)

Of the 24 trials, seven implemented moderate intensity continuous exercise (MICON) either on a cycle ergometer or on a treadmill (Chang et al. 2015; Cubbon et al. 2010; Lockard et al. 2010; Niemiro et al. 2017; Ross et al. 2018; Stromberg et al. 2017; Lansford et al. 2016) with the prescription of the intensity varying within trials and expressed as HR > 140 bpm (Chang et al. 2015), 80% of individual lactate threshold (Cubbon et al. 2010), 60–70% of VO_2max_ (Lansford et al. 2016), 75 ± 5% VO_2max_ (Lockard et al. 2010), 70% VO_2peak_ (Niemiro et al. 2017; Ross et al. 2018) and 50 – 65% VO_2max_ (Stromberg et al. 2017). The duration of the MICON protocols ranged between 30 – 60 min and one trial set it until the participants reached a total energy expenditure of 2.5 MJ (megajoules) (Lansford et al. 2016). Finally, one trial compared the responses between 30 min intense running (at 100% of individual anaerobic threshold), 30 min moderate running (at 80% IAT) and 10 min moderate running (at 80% individual anaerobic threshold) (Laufs et al. 2005).

Five trials utilised a variety of maximal exercise tests such as a VO_2max_ test (Thijssen et al. 2006; Shill et al. 2016), a cardiopulmonary exercise test (CPET) (Van Craenenbroeck et al. 2008b), a modified Bruce treadmill protocol (Yang et al. 2007) and a 1,500 m maximal field test (Bonsignore et al. 2010).

Three trials incorporated prolonged duration aerobic exercise, with two examining the responses after a marathon race (Adams et al. 2008; Bonsignore et al. 2010) and one after 240 min cycling at 70% of the individual anaerobic threshold (Mobius-Winkler et al. 2009).

Some trials investigated the responses to resistance exercise (RE) protocols with one comparing three different whole-body RE protocols (60%, 70% and 80% of 1 repetitions maximum (1RM)) (Ribeiro et al. 2017), one examining the responses of a whole-body circuit RE protocol (Ross et al. 2014) and one implementing a unilateral knee extension on an isokinetic dynamometer (Montgomery et al. 2019). When comparing the resistance training regime with different exercise regimes, one trial compared a whole-body RE protocol (75%1RM) with concentric cycling (80%VO_2max_ for 43 ± 5 min) and an eccentric downhill treadmill run (80%VO_2max_ for 51 ± 6 min) (Kruger et al. 2015).

Four trials investigated the responses to aerobic exercise with various high intensity interval training (HIIT) protocols (Kruger et al. 2016; Harris et al. 2017; Sapp et al. 2019; O'Carroll et al. 2019). The aerobic continuous protocols had a moderate intensity, and duration ranged between 30 and 45 min. Regarding HIIT protocols, two trials included three-minute intervals at an intensity between 85–95% of peak power output with 3-4 min active recovery either unloaded or at 40% of peak power output, for 27 and 30 min respectively (Kruger et al. 2016; Sapp et al. 2019). The other two trials included short-stage sprint intervals (20-30 s) with recovery between 20 s up to two minutes and a total exercise duration of 12 and 30 min respectively (Harris et al. 2017; O'Carroll et al. 2019).

The most common antibody combination to identify circulating EPCs was CD34^+^/KDR^+^ in 13 out of 24 trials (Thijssen et al. 2006; Adams et al. 2008; Bonsignore et al. 2010; Cubbon et al. 2010; Laufs et al. 2005; Lockard et al. 2010; Mobius-Winkler et al. 2009; Yang et al. 2007; Montgomery et al. 2019; Harris et al. 2017; Shill et al. 2016; Van Craenenbroeck et al. 2008b; Lansford et al. 2016) followed by the CD34^+^/KDR^+^/CD45^dim^ which has been adopted by six trials (Ross et al. 2018, 2014; Stromberg et al. 2017; Montgomery et al. 2019; Ribeiro et al. 2017; O'Carroll et al. 2019). Another six EPCs identified the following phenotypes: CD133^+^/CD34^+^/KDR^+^ (Cubbon et al. 2010; Harris et al. 2017), CD34^+^/KDR^+^/CD45^−^ (Kruger et al. 2016, 2015), CD133^+^/KDR^+^ (Mobius-Winkler et al. 2009), CD45^−^/CD34^+^/CD31^+^ (Niemiro et al. 2017), CD34^+^/CD31^+^/CD45^dim/−^ (Sapp et al. 2019) and KDR^+^/CD11b^−^/CD34^+^/AC133^+^ (Chang et al. 2015). Sixty percent of the acute trials expressed EPCs in absolute numbers (cells/mL or cells/μL) (Thijssen et al. 2006; Adams et al. 2008; Bonsignore et al. 2010; Kruger et al. 2016, 2015; Mobius-Winkler et al. 2009; Niemiro et al. 2017; Ross et al. 2018, 2014; Stromberg et al. 2017; Montgomery et al. 2019; Harris et al. 2017; Van Craenenbroeck et al. 2008b; O'Carroll et al. 2019); others expressed the results as a percentage of peripheral blood mononuclear cells (PBMNCs) (Chang et al. 2015; Yang et al. 2007), percentage of lymphocytes (Cubbon et al. 2010), percentage of leucocytes (Ribeiro et al. 2017), cells per 10^5^ events (Laufs et al. 2005; Lockard et al. 2010), cells per 500,000 events (Sapp et al. 2019), PBMNCs per 50,000 events (Lansford et al. 2016) and PBMNCs per 10^5^ events (Shill et al. 2016). Variability observed in the time points that EPCs assessed (Table 2) with 12 trials including two time points with the post-exercise sample taken from immediately post to 30 min post-exercise (Thijssen et al. 2006; Adams et al. 2008; Bonsignore et al. 2010; Cubbon et al. 2010; Laufs et al. 2005; Lockard et al. 2010; Ross et al. 2018; Yang et al. 2007; Sapp et al. 2019; Harris et al. 2017; Shill et al. 2016; Van Craenenbroeck et al. 2008b; Lansford et al. 2016). Three trials had three time points with the third sample taken from 30 min up to 24 h post-exercise (Adams et al. 2013; Bonsignore et al. 2010; Chang et al. 2015; Montgomery et al. 2019); five trials had four time points with last sample taken up to 24 h (Kruger et al. 2016; Ross et al. 2014; Stromberg et al. 2017; Ribeiro et al. 2017; O'Carroll et al. 2019) and one trial had five time points and reached up to 24 h (Kruger et al. 2015). Two trials examined the kinetics of EPC mobilisation pre-, during and post-exercise with one trial using 16 time points which reached up to 1,440 min post-exercise (Mobius-Winkler et al. 2009) and one trial with seven time points reaching up to 120 min post-exercise (Niemiro et al. 2017). Finally, 10 trials reported that the blood samples taken in a fasted state (Lockard et al. 2010; Niemiro et al. 2017; Ross et al. 2018, 2014; Montgomery et al. 2019; Sapp et al. 2019; Harris et al. 2017; Shill et al. 2016; Van Craenenbroeck et al. 2008b; Lansford et al. 2016), two trials in a non-fasted state (Thijssen et al. 2006; O'Carroll et al. 2019), two trials instructed the participants to avoid a diet rich in nitrite/nitrate (Mobius-Winkler et al. 2009; Yang et al. 2007), one trial instructed to avoid supplements such as caffeine (Kruger et al. 2016) and nine trials did not report the diet status of their participants (Adams et al. 2008; Bonsignore et al. 2010; Chang et al. 2015; Cubbon et al. 2010; Kruger et al. 2015; Laufs et al. 2005; Ribeiro et al. 2017; Stromberg et al. 2017) (Table 2).

**Table 2:** Summary of blood collection time points and fasted/non-fasted status in acute trials

Study	Number of blood samples	Time point of blood collection	Fasted/non-fasted status
Adams et al. (2004)	2	Before and immediately after race	Not reported
Bonsignore et al. (2010)	3 (For marathon race)	For marathon race: Pre, 8 ± 2 min post and 18-20 h post-race	Not reported
Bonsignore et al. (2010)	2 (For the 1500 m test)	For field test: Pre and 3-5 min Post exercise	Not reported
Chang et al. (2015)	3	Pre, 10 min post and 24 h post	Not reported
Cubbon et al. (2010)	2	Immediately before and 20 min post-exercise	Not reported
Harris et al. (2017)	2	Baseline (on different date) and 30 min Post Ex	Fasted state
Kruger et al. (2015)	5	Pre, post, 1 h post, 3 h post and 24 h post	Not reported
Kruger et al. (2016)	4	Pre, post, 3 h post and 24 h post	Avoid supplements such as caffeine
Lansford et al. (2016)	2	Pre and 5 min post Ex	4 h fast
Laufs et al. (2005)	2	Pre and 10 min post Ex	Not reported
Lockard et al. (2010)	2	Pre and 30 min post Ex	12 h fast
Mobius-Winkler et al. (2009)	16	Pre, 5 min Ex, 10 min Ex, 15 min Ex, 30 min Ex, 60 min Ex, 90 min Ex, 120 min Ex, 150 min Ex, 180 min Ex, 210 min Ex, 240 min Ex, 30 min Post Ex, 60 min post Ex, 120 min post Ex, 1440 min post Ex	Nitrite/Nitrate and antioxidant restricted diet for 48 h
Montgomery et al. (2019)	3	Pre, Post Ex and 30 min Post Ex	Fasted state
Niemiro et al. (2017)	7	Pre, 20minEx, 40minEx, 60 min Ex, 15 min post Ex, 60 min post Ex, 120 min post Ex	Overnight fast
O’Carroll et al. (2019)	4	Pre, Post Ex, 2hpost Ex and 24 h post Ex	Light breakfast with no caffeine
Ribeiro et al. (2017)	4	Pre, Post Ex, 6 h post Ex, 24 h post Ex	Not reported
Ross et al. (2018)	2	Pre and post Ex	Overnight fast
Ross et al. (2014)	4	Pre, 10 min post Ex, 2 h post Ex and 24 h post Ex	Fasted
Sapp et al. (2019)	2	Pre and Post Ex	Overnight fast (≥ 10 h)
Shill et al. (2016)	2	Pre and 3.4 ± 0.4 min post Ex	Fasted state (> 10 h)
Strömberg et al. (2017)	4	Pre, Post Ex, 30 min post Ex and 2 h post Ex	Not reported
Thijssen et al. (2006)	2	Pre and Post Ex	Light breakfast
Van Craenenbroeck et al. (2008a, b)	2	Pre and 10 min Post Ex	Overnight fast
Yang et al. (2007)	2	Pre and 30 min Post Ex	Nitrite/Nitrate restricted diet for 48 h

### Chronic trial characteristics and intervention details

The nine trials which examined the chronic effects of exercise on circulating EPCs included 258 participants with 62.4% males and 37.6% females and a large age range between 22 and 76 years (Table 3). Four trials included both males and females (Landers-Ramos et al. 2016; Rakobowchuk et al. 2012; Niemiro et al. 2018; Cesari et al. 2012), four included only male individuals (Thijssen et al. 2006; Tsai et al. 2016; Xia et al. 2012; Yang et al. 2013) and one included only females (Jo et al. 2019).

**Table 3:** Summary of studies examining the chronic effects of exercise on EPCs

Study	Study design	Participant characteristics	Exercise Prescription	EPC phenotype identified by flow cytometry and units in brackets	Results on circulating EPCs and other major findings
**Trials that included MICON exercise**					
Cesari et al.(2012)	Independent groups, before and after	**Compliant group:** n = 21 healthy overweight/obese **Non-compliant group:** n = 19 healthy overweight/obese Median age **(whole group):** n = 40, 48yrs, 55% males	**Length:** 3 months **Frequency:** 3x/Week. **Duration:** 45 min. **Modality:** Aerobic exercise (walking/running) **Intensity:** ≤ IAT	CD34^+^/KDR^+^ and CD133^+^/KDR^+^ and CD34^+^/CD133^+^/KDR^+^ (Cells/10^6^ events)	**↑ in compliant group only** **Compliant group:** CD34^+^/KDR^+^: Pre 14 (9–19) vs Post 21 (15–27), *P* = 0.04 CD133^+^/KDR^+^: Pre 13 (8–18) vs Post 20 (15–24), *P* = 0.02.—CD34^+^/CD133^+^/KDR^+^: Pre 9 (6–13) vs Post 14 (11–17), *P* = 0.04 **Non-compliant group:** CD34^+^/KDR^+^: Pre 18 (12–23) vs Post 21 (13–28), *P* = 0.3. CD133^+^/KDR^+^: Pre 18 (12–24) vs Post 21 (14–28), *P* = 0.2.—CD34^+^/CD133^+^/KDR^+^: Pre 15 (11–18) vs Post 18 (12–24), *P* = 0.2. Relationship between fat mass loss and increase in CD133^+^/KDR^+^ EPCs in compliant group (r = 0.50, *P* = 0.04)
Landers-Ramos et al. (2016)	Single arm	**EX group:** n = 11 healthy, 36% males, 61.0 ± 2.1yrs, VO_2max_: 29.3 ± 1.5 ml.kg^1^.min^−1^	**Length:** 10 days. **Frequency:** Daily. **Duration:** 60 min/day. **Modality:** Aerobic exercise (walking/running). **Intensity:** ~ 70%VO_2max_	CD34^+^/KDR^+^ (Cells / 10^5^ events)	**↑ by 104%. Pre: 24.86 ± 6.55 vs Post 50.78 ± 12.96, *P* < 0.05** ↔ VO_2max;_ ↑ FMD; no correlation between ΔFMD and ΔEPCs (*P* > 0.05)
Niemiro et al. (2018)	Independent groups, before and after	**EX group 1:** n = 17 healthy lean sedentary, 53% males, 23.9 ± 5.4yrs, VO_2max_: 40.3 ± 5.3 ml.kg^−1^.min^−1^ **EX group 2:** n = 10 healthy obese sedentary, 30% males, 29.0 ± 8.0yrs, VO_2max_: 31.3 ± 5.0 ml.kg^−1^.min^−1^	**Length:** 6 weeks. **Frequency:** 3x/Week. **Duration:** 30-60 min. **Modality:** Aerobic exercise on treadmill or bike. **Intensity:** 60–75% HRR	CD45^−^/CD34^+^/CD31^+^ (Cells/μl)	** ↔ in both groups after intervention** **EX group 1:** Pre 0.01 ± 0.02 vs Post 0.005 ± 0.005, *P* > 0.05 **EX group 2:** Pre 0.05 ± 0.012 vs Post 0.008 ± 0.01, *P* > 0.05 **↑** VO_2max_ (both groups); ↓ SDF-1α (EX group 2); ↔ G-CSF
Thijssen et al. (2006)	Single arm	**EX group:** n = 8 healthy sedentary, 100% males, 67-76yrs, VO_2max_: 30.8 ± 4.8 ml.kg^−1^.min^1^	**Length:** 8 weeks. Frequency: 3x/Week. **Duration:** 20 min. **Modality:** Aerobic exercise on a cycle ergometer. **Intensit**y: 65%HRR up to 85%HRR	CD34^+^/KDR^+^ (Cells/μl)	**↓ by 45.7% post training** Pre 35 ± 12 vs Post 19 ± 8, *P* < 0.05 ↔ VO_2max_; ↓ VEGF
Xia et al. (2012)	Single arm	**EX group:** n = 25 healthy elderly, 100% males, 67.8 ± 3.4yrs	**Length:** 12 weeks. **Frequency:** 3x/Week. **Duration:** 30 min. **Modality:** Aerobic exercise on treadmill. **Intensity:** Not reported	CD34^+^/KDR^+^ and CD133^+^/KDR^+^ (% PBMNCs)	**↑ in both EPC phenotypes** CD34^+^/KDR^+^: Pre 0.023 ± 0.005 vs Post 0.039 ± 0.006, *P* < 0.05 CD133^+^/KDR^+^: Pre 0.019 ± 0.004 vs Post 0.027 ± 0.01, *P* < 0.05 **↑** FMD
Yang et al. (2013)	Independent groups, before and after	**EX group 1**: n = 10 healthy elderly sedentary, 100% males, 61 ± 3yrs **EX group 2:** n = 10 healthy young sedentary, 100% males, 27 ± 3yrs	**Length:** 12 weeks. **Frequency:** 3x/Week. **Duration:** 30 min. **Modality:** Aerobic exercise on a treadmill. **Intensity**: 4.0 METS for older sedentary and 5.5 METS for young sedentary	CD34^+^/KDR^+^ (% PBMNCs)	**↑ by ~ 83% and ~ 22% for EX group 1 and 2 respectively** **EX group 1:** Pre 0.018 ± 0005 vs Post 0.033 ± 0.010, *P* < 0.005. **EX group 2:** Pre 0.027 ± 0.006 vs Post 0.033 ± 0.006, *P* < 0.005 **↓** baPWV by 5.1% and 2.6% for EX group 1 and 2 respectively; relationship between decreased baPWV with EPCs (r = 0.73, *P* < 0.05)
**Trials that included HIIT**					
Rakobowchuk et al. (2012)	Independent groups, before and after	**EX group 1:** n = 9 healthy, 33% males, 23.7 ± 3.4yrs, VO_2peak_: 34.7 ± 6.5 ml.kg^−1^.min^1^ **EX group 2:** n = 11 healthy, 36% males, 23.1 ± 2.5yrs, VO_2peak_: 35.7 ± 8.3 ml.kg^−1^.min^1^	**Length:** 6 weeks. **Frequency:** 3x/week. **Duration:** Progressed from 30 to 40 min/session. **Modality/Intensity:** **EX group 1**: Repeated 10 s interval at 120% PWR with 20 s recovery at 20 W. **EX group 2:** Repeated 30 s intervals at 120% PWR with 60 s recovery at 20 W	CD34^+^/CD133^+^/KDR^+^ and CD34^+^/KDR^+^ (Cells/mL)	** ↔ in any group** **EX group 1:** CD34^+^/CD133^+^/KDR^+^: Pre 16 ± 18 vs Post 14 ± 12, *P* > 0.05. CD34^+^/KDR^+^: Pre 67 ± 80 vs Post 47 ± 47, *P* > 0.05 **EX group 2:** CD34^+^/CD133^+^/KDR^+^: Pre 8 ± 6 vs Post 19 ± 13, *P* > 0.05. CD34^+^/KDR^+^: Pre 34 ± 37 vs Post 79 ± 125, *P* > 0.05 **↑** VO_2max_ (EX group 2); ↔ FMD (both groups)
**Trials that compared HIIT to MICON**					
Tsai et al.(2016)	RCT	**EX group 1:** n = 20 healthy sedentary, 100% males, 22.2 ± 1.6yrs, VO_2max_: 43.2 ± 1.2 ml.kg^−1^.min^1^ **EX group 2:** n = 20 healthy sedentary, 100% males, 22.3 ± 1.0yrs, VO_2max_: 40.3 ± 1.4 ml.kg^−1^.min^1^ **Control group**: n = 20 healthy sedentary, 100%males, 22.4 ± 2.1yrs, VO_2max_: 42.3 ± 2.1 ml.kg^−1^.min^1^	**Length:** 6 weeks. **Frequency:** 5x/Week. **Duration:** 30 min. **Modality/Intensity:** **EX group 1:** HIIT cycle ergometer 5 sets of 3 min at 80%VO_2peak_ with 3 min recovery at 40%VO_2peak_ **EX group 2:** Aerobic exercise on a cycle ergometer at 60%VO_2peak_	CD34^+^/KDR^+^ and CD34^+^/KDR^+^/CD133^+^ and CD34^+^/KDR^+^/CD31^+^ (Cells/mL)	**↑ for both EPC phenotypes in EX group 1 and 2 but no in control group** **EX group 1:** CD34^+^/KDR^+^: Pre 422 ± 54 vs Post 565 ± 82, *P* < 0.05. CD34^+^/KDR^+^/CD133^+^: Pre 196 ± 41 vs Post 298 ± 79, *P* < 0.05.—CD34^+^/KDR^+^/CD31^+^: Pre 76 ± 19 vs Post 169 ± 39, *P* < 0.05 **EX group 2:** CD34^+^/KDR^+^: Pre 400 ± 42 vs Post 587 ± 79, *P* < 0.05. CD34^+^/KDR^+^/CD133^+^: Pre 185 ± 38 vs Post 278 ± 71, *P* < 0.05. CD34^+^/KDR^+^/CD31^+^: Pre 66 ± 11 vs Post 109 ± 36, *P* > 0.05 **Control group:** CD34^+^/KDR^+^: Pre 432 ± 54 vs Post 426 ± 63, *P* > 0.05. CD34^+^/KDR^+^/CD133^+^: Pre 167 ± 31 vs Post 177 ± 36, *P* > 0.05 CD34^+^/KDR^+^/CD31^+^: Pre 72 ± 10 vs Post 79 ± 16, *P* > 0.05 **↑** Mon-1 EPCs (EX group 1); **↑** Mon-2 EPCs (EX group 1); **↑** VO_2max_ (both EX groups but higher in EX group 1); **↑** NOx (both EX groups but higher in EX group 1); **↑** plasma VEGF-A (EX group 1); **↑** SDF-1α (EX group 1) **↑**; MMP-9 (EX group 1); correlation between VO_2max_ with CD34^+^/KDR^+^/CD133^+^ EPCs, (r = 0.673, P < 0.0001)
**Trials that compared MICON exercise and other forms of aerobic exercise**					
Jo et al. (2019)	RCT	**EX group 1:** n = 21 post-menopausal with CV risk: > 20%, 100% females, > 50yrs, VO_2peak_: 22.3 ± 0.68 ml.kg^1^.min^1^ **EX group 2:** n = 13 post-menopausal with CV risk: > 20%, 100% females, > 50yrs, VO_2peak_: 23.0 ± 0.69 ml.kg^−1^.min^−1^ **Control:** n = 13 post-menopausal with CV risk: > 20%, 100% females, > 50yrs, VO_2peak_:21.0 ± 0.6 ml.kg^−1^.min^−1^	**EX group 1: Length:** 12 weeks. **Frequency:** daily. **Duration:** 40 min. **Modality:** Aerobic exercise (Running based exergame). **Intensity:** 120 ± 19 bpm. **EX group 2: Length:** 12 weeks. **Frequency:** daily. **Duration:** 40 min. **Modality:** Aerobic exercise (Running/jogging). **Intensity:** 60–80%HRR	CD34^+^/KDR^+^ (Cells/μl)	**↑ of CD34**^**+**^**/KDR**^**+**^** EPCs in EX group 2 only. ** **EX group 1:** Pre 13.8 ± 35 vs Post 17.1 ± 22.2, *P* > 0.05 **EX group 2:** Pre 1.5 ± 3.8 vs Post 19.2 ± 17.5, *P* < 0.01 **Control group:** Pre 6.9 ± 9.5 vs Post 6.2 ± 11.9, *P* > 0.05 **↑** VO_2peak_ (EX groups 1 and 2); **↑** FMD (EX groups 1 and 2)

*baPWV* branchial artery pulse wave velocity, *bpm* beats per minute, *CV* cardiovascular, *EPCs* endothelial progenitor cells, *EX* exercise, *FMD* flow mediated dilatation, *G-CSF *granulocyte colony stimulating factor, *HIIT* high intensity interval training, *HRR* heart rate reserve, *IAT* individual anaerobic threshold, *mL* millilitre, *METS *metabolic equivalents, *MICON* moderate intensity continuous training, *MMP-9 *matrix metalloproteinase 9, *Mon-1 EPCs *Monocytic 1 derived EPCs, *Mon-2 EPCs *Monocytic 2 derived EPCs, *NOx* Nitric oxide metabolites nitrite/nitrate, *PBMNCs* peripheral blood mononuclear cells, *PWR* peak work rate, *RCT* randomised control trial, *SDF-1α* stromal cell derived factor 1 alpha, *VEGF* vascular endothelial growth factor, *VO*_*2peak/max*_ peak/max oxygen uptake, *W* watts, *yrs* years, *μL* microliter, ↑ indicates significant increase, ↓ indicates significant reduction ↔ indicates no significant change

The most common exercise modality employed was MICON exercise with the length of the intervention varying between 10 days to 12 weeks (Thijssen et al. 2006; Landers-Ramos et al. 2016; Niemiro et al. 2018; Xia et al. 2012; Yang et al. 2013; Cesari et al. 2012). The most common weekly exercise frequency of the trials was three times per week with one trial prescribing daily exercise (Landers-Ramos et al. 2016). The duration of the sessions ranged from 20 to 60 min. The intensity prescribed was based on the IAT (Cesari et al. 2012), 70% of VO_2max_ (Landers-Ramos et al. 2016), 65 – 85% heart rate reserve (Thijssen et al. 2006), 60 – 75% of heart rate reserve (Niemiro et al. 2018), 5.5 metabolic equivalents for young group and 4.0 metabolic equivalents for older group respectively (Yang et al. 2013) and one trial did not report intensity (Xia et al. 2012). Regarding other forms of exercise that have been employed, one trial compared a MICON protocol (60% VO_2peak_) with a time matched HIIT protocol (5 × 3 min at 80% VO_2peak_ with 3 min recovery at 40% VO_2peak_) (Tsai et al. 2016). The participants in both protocols exercised for six weeks, three times per week. Another trial compared a short sprint interval exercise (10 s at 120% peak work rate with 20 s recovery at 20Watts) with a long sprint interval exercise (30 s at 120% peak work rate with 60 s recovery at 20Watts) for six weeks, three times per week (Rakobowchuk et al. 2012). Another trial compared a traditional MICON protocol (60–80% of heart rate reserve) with aerobic exercise based on exergame (Jo et al. 2019), which is an alternative exercise modality based on an interactive video game (Graves et al. 2007).

Eight out of nine trials quantified circulating EPCs using the CD34^+^/KDR^+^ antibody combination (Thijssen et al. 2006; Jo et al. 2019; Landers-Ramos et al. 2016; Rakobowchuk et al. 2012; Tsai et al. 2016; Xia et al. 2012; Yang et al. 2013; Cesari et al. 2012) and one trial used CD45^−^/CD34^+^/CD31^+^ (Niemiro et al. 2018). Another three EPC phenotypes identified in trials that used more than one EPC phenotype: CD133^+^/KDR^+^ (Xia et al. 2012; Cesari et al. 2012), CD34^+^/CD133^+^/KDR^+^ (Rakobowchuk et al. 2012; Tsai et al. 2016; Cesari et al. 2012) and CD34^+^/KDR^+^/CD31^+^ (Tsai et al. 2016). Most of the trials expressed EPCs as an absolute number (cells/mL or cells/μL) (Sandri et al. 2016; Thijssen et al. 2006; Jo et al. 2019; Rakobowchuk et al. 2012; Niemiro et al. 2018; Tsai et al. 2016), two trials expressed them as a percentage of PBMNCs (Xia et al. 2012; Yang et al. 2013), one as cells per 10^6^ events (Cesari et al. 2012) and one as cells per 10^5^ events (Landers-Ramos et al. 2016). All trials included two blood sample time points, one pre-intervention and one post-intervention (Table 4). However, only three trials reported when the post-intervention sample was taken, which had a range between 24 and 96 h (Landers-Ramos et al. 2016; Rakobowchuk et al. 2012; Niemiro et al. 2018). Six out of the ten trials reported that blood samples were taken in a fasted state (Jo et al. 2019; Landers-Ramos et al. 2016; Rakobowchuk et al. 2012; Niemiro et al. 2018; Tsai et al. 2016; Cesari et al. 2012), one in a non-fasted state (Thijssen et al. 2006) and two did not report it (Xia et al. 2012; Yang et al. 2013) (Table 4).

**Table 4:** Summary of blood collection time points and in fasted/non-fasted state in chronic trials

Study	Number of blood collections	Time point of blood collections	Food intake condition during the blood sampling
Cesari et al. (2012)	2	Pre and Post intervention	Overnight fast
Jo et al. (2019)	2	Pre and Post intervention	8 h fasting
Landers-Ramos et al. (2016)	2	Pre and Post intervention (24 h after the last training session)	12 h fasting
Niemiro et al. (2018)	2	Pre and Post intervention (3–4 days after the last training session)	8-10 h fasting
Rakobowchuk et al. (2012)	2	Pre and Post intervention (between 48 and 72 h after the last training session)	8-12 h fasting
Thijssen et al. (2006)	2	Pre and Post intervention	Light breakfast
Tsai et al. (2016)	2	Pre and Post intervention	8 h fast
Xia et al. (2012)	2	Pre and Post intervention	Not reported
Yang et al. (2013)	2	Pre and Post intervention	Not reported

### Acute effects on circulating EPCs and angiogenic factors

Table 1 presents the acute effects on circulating EPCs and angiogenic factors. From the trials that evaluated the acute effects of MICON exercise on EPCs, three trials reported significant increases (Cubbon et al. 2010; Chang et al. 2015; Ross et al. 2018) at various time points post-exercise and four reported no significant changes (Lockard et al. 2010; Niemiro et al. 2017; Stromberg et al. 2017; Lansford et al. 2016). Regarding the responses of pro-angiogenic factors, two trials measured VEGF (Ross et al. 2018; Stromberg et al. 2017) where one found an increase (Ross et al. 2018) and the other did not (Stromberg et al. 2017). Of the four trials that measured SDF-1α (Chang et al. 2015; Niemiro et al. 2017; Ross et al. 2018; Stromberg et al. 2017), two found an increase (Chang et al. 2015; Niemiro et al. 2017) and two did not report significant changes (Ross et al. 2018; Stromberg et al. 2017). One trial measured stem cell factor (SCF) and found a significant increase post-exercise (Niemiro et al. 2017). Finally, Laufs et al. (2005) reported that only the intensive running (30 min) and longer moderate intensity running (30 min) elicited significant increases in CD34^+^/KDR^+^ EPCs, but not the short duration moderate intensity running (10 min). However, despite the changes in EPCs no change was reported for serum VEGF and cortisol.

From the five trials that implemented maximal exercise, four reported significant increases in EPC numbers (Bonsignore et al. 2010; Yang et al. 2007; Shill et al. 2016; Van Craenenbroeck et al. 2008b) and one reported no changes (Thijssen et al. 2006). From the four trials that reported increases in EPC numbers, three measured VEGF and all found no changes after exercise (Bonsignore et al. 2010; Yang et al. 2007; Van Craenenbroeck et al. 2008b). In addition, one measured VEGF-C, VEGF-D, angiopoietin one, angiopoietin two and SCF and found increases in VEGF-C, SCF and Ang-2 only (Bonsignore et al. 2010). One measured granulocyte macrophage colony stimulating factor and found no changes (Yang et al. 2007), while two measured plasma Nitrite/Nitrate (Yang et al. 2007; Van Craenenbroeck et al. 2008b) with one reporting a significant increase (Yang et al. 2007) which was correlated with the increase in CD34^+^/KDR^+^ EPCs (*r* = 0.70, *P* < 0.05) and the other reported no changes (Van Craenenbroeck et al. 2008b). Thijssen et al. (2006), despite not finding any changes in EPCs, reported an increase in VEGF levels in the sedentary young group compared to trained young and sedentary old groups.

In the trials that incorporated prolonged duration exercise the results were equivocal after a marathon race. One trial (Bonsignore et al. 2002) reported nearly a two-fold increase in CD34^+^/KDR^+^ EPCs which was accompanied by increases in IL-6, VEGF-C, Ang-1, Ang-2 and SCF but with no changes in VEGF-A and VEGF-D. Another trial showed no changes in EPCs with a reduction on plasma VEGF (Adams et al. 2008) while another (Mobius-Winkler et al. 2009) reported that both CD34^+^/KDR^+^ and CD133^+^/KDR^+^ EPCs reached a peak of 5.5-fold increase at 240 min and 3.5-fold increase at 210 min respectively, following a bout of cycling; these were accompanied by a 1.9-fold increase in serum VEGF and 16.5-fold increase in IL-6. More important the ΔCD133^+^/KDR^+^ EPCs was positively correlated with the ΔVEGF (r = 0.67, *P* = 0.0045) while no relationship was observed between ΔCD34^+^/KDR^+^ EPCs and ΔVEGF.

From the trials that compared a HIIT with a MICON protocol, one (Kruger et al. 2016) found a significant increase in CD34^+^/KDR^+^/CD45^−^ EPCs in both protocols with no significant difference between them, while another trial reported a significant reduction in CD34^+^/CD31^+^/CD45^dim/−^ EPCs for both modalities (Sapp et al. 2019). The other two trials that compared short-stage sprint interval protocols vs a MICON protocols reported no changes in circulating EPCs (Harris et al. 2017; O'Carroll et al. 2019).

Regarding RE protocols, two trials reported significant increase of EPCs (Ross et al. 2014; Montgomery et al. 2019) with one of them (Ross et al. 2014) reporting a significant elevation of several pro-angiogenic factors such as serum VEGF-A, VEGF-C, VEGF-D, granulocyte colony stimulating factor (G-CSF), matrix metalloproteinase 2 and 9 at various time points. Moreover, Ribeiro et al. (Ribeiro et al. 2017) reported that the largest increase of CD34^+^/CD309^+^/CD45^dim^ EPCs, was after the whole-body RE at 80%1RM compared to 70% and 60%1RM in young women. All three protocols elicited increases in plasma VEGF, plasma erythropoietin, with an increase in hypoxic inducible factor one alpha (HIF-1α) being observed only after the 70% and 80%1RM RE protocols. When data are pooled together there is a positive relationship between change in EPCs and VEGF (*r* = 0.492, *P* = 0.002) and HIF-1α (*r* = 0.388, *P* = 0.016) from baseline to 6 h post-exercise, respectively. No changes in plasma SDF-1α were reported. Finally, Kruger et al.(Kruger et al. 2015) reported that a RE, a concentric endurance and an eccentric endurance protocol all induced EPC mobilisation which was time dependant. Following the RE protocol, EPC levels were elevated for up to 24 h, and up to 48 h after the eccentric endurance protocol. Moreover, all three protocols induced an elevation of G-CSF which was positively correlated with EPCs at 3 h post-exercise (*r* = 0.54, *P* < 0.05).

### Chronic effects on EPCs, fitness status, angiogenic factors, and endothelial function

Table 3 shows the chronic effects of exercise on EPCs, fitness status, angiogenic factors, and endothelial function in healthy populations. The six trials that incorporated MICON exercise varied because of the different populations recruited. Two trials (Niemiro et al. 2018; Cesari et al. 2012) focussed on overweight/obese individuals, where one (Cesari et al. 2012) found that the overweight compliant group had a significant increase in EPCs after the intervention whereas the non-compliant group did not. Interestingly, in the compliant group there was a positive relationship between fat mass loss and increase in CD133^+^/KDR^+^ EPCs (*r* = 0.50, *P* = 0.04). In contrast, Niemiro et al. (2018) found no change in CD45^−^/CD34^+^/CD31^+^ EPCs both in healthy obese and healthy lean individuals. Those results were accompanied by a lack of change in plasma G-CSF, while plasma SDF-1α significantly reduced in the healthy obese group. The other four trials focussed on aged individuals (> 60 years old) with (Yang et al. 2013) or without (Thijssen et al. 2006; Landers-Ramos et al. 2016; Xia et al. 2012) a young control group. Yang et al. (2013) found a significant increase in CD34^+^/KDR^+^ EPCs in both groups. However, the increase in circulating EPCs was more pronounced in the elderly versus the young group (~ 83% vs ~ 22%). The improvement in EPC levels was accompanied by an improvement in arterial stiffness measured by brachial artery pulse wave velocity, which was positively correlated with ΔEPCs (*r* = 0.73, *P* < 0.05). Similarly, another trial (Xia et al. 2012) reported significant improvements both in EPC numbers and FMD in healthy elderly individuals. Another trial (Landers-Ramos et al. 2016), despite the short duration of the intervention (10 days), resulted in a 104% increase in EPCs with concurrent improvements in FMD. However, the latter was not correlated with the increase in EPCs. In contrast with the above findings, Thijssen et al. (2006) reported a reduction in EPCs and plasma VEGF levels with no significant improvement in VO_2max_ following 8 weeks of vigorous intensity aerobic exercise (cycle ergometry) performed for 20 min, 3 times weekly by 8 sedentary older men. The trial (Jo et al. 2019) that incorporated either exergame or MICON exercise in post-menopausal women found that CD34^+^/KDR^+^ EPCs increased only after the latter protocol. Both groups equally resulted in an improvement in VO_2peak_ and FMD, while those changes were not observed in the control group.

Regarding the trials that examined the chronic effects of HIIT, a trial (Rakobowchuk et al. 2012) reported no change in EPC numbers and FMD after short (10 s) or longer (30 s) stage HIIT, despite the improvement in VO_2max_ in both protocols. In contrast, another trial (Tsai et al. 2016) reported that both a HIIT and MICON protocol resulted in significant increases in CD34^+^/KDR^+^ and CD34^+^/KDR^+^/CD133^+^ EPCs. However, CD34^+^/KDR^+^/CD31^+^ EPCs increased only in the HIIT group along with monocyte derived EPCs. Both protocols increased plasma nitrite/nitrate with a more pronounced increase after the HIIT protocol. Moreover, only the HIIT protocol led to an increase in pro-angiogenic factors such as VEGF-A, SDF-1α and MMP-9. Finally, the authors reported a positive correlation between VO_2max_ and CD34^+^/KDR^+^/CD133^+^ EPCs post-intervention (r = 0.673, *P* < 0.0001).

### Study quality assessment

Seven trials were assessed with the TESTEX scale (Sapp et al. 2019; Kruger et al. 2016; Harris et al. 2017; O'Carroll et al. 2019; Laufs et al. 2005; Jo et al. 2019; Tsai et al. 2016). The average score was 9.7 ± 1.4 out of 15, ranging from 7 – 11 (Table 5). Only two trials reported randomisation details (Sapp et al. 2019; Kruger et al. 2016). The majority of the trials lost points in allocation concealment and blinding of the assessor. Intention to treat analysis was not considered in any trial.

**Table 5:** Quality assessment scores based on TESTEX scale

Study	Study quality criteria					Study reporting criteria							TESTEX score (out of 15)
	Eligibility criteria specified	Randomisation specified	Allocation concealment	Groups similar at baseline	Blinding of assessor	Outcome measures assessed in 85% of patients	Intention to treat analysis	Between group statistical comparisons reported	Point measures and measures of variability for all reported outcome measures	Activity monitoring in control groups	Relative exercise intensity remained constant	Exercise volume and energy expenditure	
Sapp et al. (2019)	1	1	0	1	0	2	0	2	1	1	1	1	11
Kruger et al. (2016)	1	1	0	1	0	2	0	2	1	1	1	1	11
Harris et al.(2017)	1	0	0	1	0	2	0	2	1	1	1	1	10
Jo et al. (2019)	1	0	0	1	1	2	0	2	1	1	0	1	10
Tsai et al. (2016)	1	0	0	1	0	2	0	2	1	1	1	1	10
O’Carroll et al. (2019)	0	0	0	1	0	2	0	2	1	1	1	1	9
Laufs et al. (2005)	1	0	0	1	0	1	0	0	1	1	1	1	7

The trials that included independent groups were assessed with the observational cohort and cross-sectional studies tool (Table 6). All 15 of these trials were classified as “good”. Blinding of the assessors for the analysis of EPCs as a main outcome could not be determined in most of the included trials.

**Table 6:** Quality assessment of observational cohort and cross-sectional trials

Study	Research question	Specified inclusion criteria	Participation ≥ 50%	Uniform eligibility criteria	Sample size	Exposure assessment prior to outcome measure	Sufficient timeframe for effect	Different levels of the exposure of interest	Exposure measure and assessment	Repeated exposure assessment	Outcome measures	Blinding	Follow up rate	Statistical analyses	Quality rating
Bosignore et al. (2010) (Marathon race trial)	Y	CD	Y	Y	CD	Y	N	Y	Y	N	Y	Y	Y	Y	Good
Bosignore et al. (2010) (1500 m trial)	Y	CD	Y	Y	CD	Y	N	Y	Y	N	Y	Y	Y	Y	Good
Cubbon et al. (2010)	Y	Y	Y	Y	Y	Y	N	N	Y	N	Y	CD	N/A	Y	Good
Kruger et al. (2015)	Y	CD	Y	CD	N	Y	Y	Y	Y	N	Y	CD	N/A	Y	Good
Lansford et al. (2016)	Y	Y	Y	CD	Y	Y	N	N	Y	N	Y	CD	N/A	Y	Good
Lockard et al. (2010)	Y	Y	Y	CD	N	Y	Y	N	Y	N	Y	CD	N/A	Y	Good
Ribeiro et al. (2017)	Y	Y	Y	Y	N	Y	Y	Y	Y	N	Y	CD	N/A	Y	Good
Ross et al. (2018)	Y	CD	Y	CD	Y	Y	N	N	Y	N	Y	CD	N/A	Y	Good
Shill et al. (2016)	Y	Y	Y	CD	N	Y	N	N	Y	N	N	CD	N/A	Y	Good
Thijssen et al. (2006)	Y	Y	Y	Y	Y	Y	N	N	Y	N	Y	CD	N/A	Y	Good
Van Craenenbroeck et al. (2008a, b)	Y	Y	Y	Y	Y	Y	N	N	Y	N	Y	CD	N/A	Y	Good
Cesari et al. (2012)	Y	Y	Y	CD	Y	Y	Y	N	Y	N	Y	CD	N/A	Y	Good
Yang et al. (2013)	Y	Y	Y	Y	Y	Y	Y	N	Y	N	Y	CD	N/A	Y	Good
Niemiro et al.(2018)	Y	Y	Y	Y	CD	Y	Y	N	Y	N	Y	CD	N/A	Y	Good
Rakobowchuk et al. (2012)	Y	Y	Y	Y	N	Y	Y	Y	Y	N	Y	CD	Y	Y	Good

*CD* cannot decide, *N* no, *NA* not applicable, *Y* Yes

Eleven single arm trials were assessed with the “Before-After (Pre-Post) studies with no control group” appraisal tool (Table 7). Of those, nine were classified as “good” (Adams et al. 2008; Mobius-Winkler et al. 2009; Stromberg et al. 2017; Niemiro et al. 2017; Ross et al. 2014; Yang et al. 2007; Landers-Ramos et al. 2016; Thijssen et al. 2006; Montgomery et al. 2019) and two as “fair” (Chang et al. 2015; Xia et al. 2012). Group level intervention and individual level outcome efforts criteria did not apply to any included trial.

**Table 7:** Risk of bias for before-after (Pre-Post) trials with no control group

Study	Clear research question	Specified inclusion criteria	Participants representative	Enrolment of all eligible participants	Sample size	Clear description of intervention	Definition, validity, reliability of outcome measures	Blind outcome assessment	Follow up rate	Statistical analysis	Multiple outcome measures	Group level intervention & individual outcome effort	Quality rating
Adams et al. (2008)	Y	Y	Y	Y	CD	Y	Y	Y	Y	Y	Y	NA	Good
Mobius-Winkler et al. (2009)	Y	Y	Y	Y	CD	Y	Y	CD	Y	Y	Y	NA	Good
Niemiro et al.(2017)	Y	Y	N	Y	CD	Y	Y	CD	Y	Y	Y	NA	Good
Ross et al. (2014)	Y	Y	Y	Y	CD	Y	Y	CD	Y	Y	Y	NA	Good
Strömberg et al. (2017)	Y	Y	Y	Y	Y	Y	Y	CD	Y	Y	Y	NA	Good
Yang et al. (2007)	Y	Y	N	Y	CD	Y	Y	CD	Y	Y	Y	NA	Good
Landers-Ramos et al. (2016)	Y	Y	D	CD	Y	Y	Y	CD	Y	Y	Y	NA	Good
Thijssen et al. (2006)	Y	Y	CD	Y	Y	Y	Y	CD	Y	Y	Y	NA	Good
Montgomery et al. (2019)	Y	Y	CD	Y	Y	Y	Y	CD	Y	Y	Y	NA	Good
Chang et al. (2015)	Y	Y	N	CD	N	Y	Y	CD	Y	Y	Y	NA	Fair
Xia et al. (2012)	Y	Y	Y	CD	CD	N	Y	CD	Y	Y	Y	NA	Fair

*CD* cannot decide, *NA* not applied, *N* no, *Y* yes

## Discussion

The primary aim of this systematic review was to dissect the acute and chronic effects of different forms of exercise on the number of EPCs in healthy adult populations. A secondary aim was to delineate the mechanism of exercise-induced mobilisation on circulating EPCs via the analysis of several circulating pro-angiogenic factors and identify links with vascular function and cardiorespiratory fitness.

In the trials that investigated the acute effects of exercise on circulating EPCs, the different forms of exercise were identified were: MICON, HIIT, maximal exercise, RE, prolonged, eccentric and concentric endurance. It was observed that different exercise protocols induced different responses in circulating EPCs. Prolonged endurance exercise and RE provide a longer lasting elevation of circulating EPCs, followed by maximal exercise. Some evidence exists that eccentric and concentric vigorous endurance exercise induces increases on EPCs as well, whereas MICON protocols produced equivocal findings. Short-stage HIIT (< 30 s) protocols were shown to have no effects on EPC mobilisation, with long-stage HIIT (3 min bouts) seeming to induce increases or decreases in EPC levels post-exercise. Regarding the mechanism of acute exercise-induced EPC mobilisation the most studied pro-angiogenic factor was VEGF. Moreover, there was evidence that nitric oxide (NOx) availability, chemokines (SDF-1α) and cytokines such as IL-6 seem to play a role in exercise-induced EPC mobilisation which is dependent on the exercise form.

In the trials which examined the chronic effects of exercise on circulating EPCs, the most studied form of exercise was a MICON protocol. There was strong evidence that exercise using a MICON protocol, 10 days up to 3 months, increases EPC numbers in older healthy adults and this was accompanied by improvements in FMD and baPWV. Regarding HIIT exercise, evidence showed that short-stage HIIT intervention does not induce any changes in EPCs whereas long-stage HIIT (4 min bouts) appears to be superior when compared to a MICON protocol. The evidence about the exercise-induced EPC mobilisation after chronic MICON exercise was limited due to the lack of analysis of pro-angiogenic factors in the included trials. In contrast, there is evidence that the long-stage HIIT increased several pro-angiogenic factors (VEGF, MMP-9, SDF-1α) and enhanced NOx availability.

### Acute effects of exercise on EPCs

When we examined the acute effects of prolonged endurance exercise the majority of the trials (Bonsignore et al. 2010; Mobius-Winkler et al. 2009) showed large increases in circulating EPCs irrespective of the form of exercise (cycling or running). In addition, long lasting effects were induced after 4 h of cycling which extended up to 210 min for CD133^+^/KDR^+^ and 240 min for CD34^+^/KDR^+^ EPCs (Mobius-Winkler et al. 2009). However, Adams et al. (2008) failed to detect an increase on CD34^+^/KDR^+^ EPCs after a marathon race. The lack of increase in EPCs may be related to the older mean age (57 ± 6 yrs) compared to the other two trials (43.6 ± 11yrs (Bonsignore et al. 2010) and 32.4 ± 2.3 yrs (Mobius-Winkler et al. 2009)). Ageing is known to negatively affect EPC numbers (Thijssen et al. 2006; Yang et al. 2013; Ross et al. 2018). It has previously been shown that resting levels of EPCs are significantly reduced in old endurance-trained individuals compared to their younger counterparts (Yang et al. 2013). Moreover, ageing and prolonged exercise are factors inducing β receptor desensitisation (Schocken and Roth 1977; Scarpace et al. 1991; Hart et al. 2006), and it has been shown that β2 adrenergic receptor stimulation elicits an increase in EPC numbers and function while enhancing their angiogenic capacity both in vitro and in vivo (Galasso et al. 2013). This further supports that ageing combined with prolonged exercise may, at least partly, be responsible for the lack of changes in circulating EPCs in older marathon runners caused by the lack of β2 adrenergic receptor stimulation.

Prolonged vigorous endurance exercise elicits a strong stimulus for immune changes (Nielsen and Lyberg 2004), increases in several pro- and anti-inflammatory markers (Barros et al. 2017) and markers of endothelial dysfunction (Jee and Jin 2012). In the present systematic review, it has been found that IL-6 increased in the same time manner as circulating EPCs (Mobius-Winkler et al. 2009; Bonsignore et al. 2010). The potential role of IL-6 as a pro-angiogenic factor has been demonstrated by its role in the stimulation of EPC proliferation, migration and tube formation in a dose dependent manner (Fan et al. 2008). It has been suggested that VEGF is upregulated under ischaemic conditions and acts synergistically with SDF-1α to promote EPC mobilisation (Petit et al. 2007; Tilling et al. 2009). However, the results from the trials that used prolonged endurance exercise were equivocal since only one trial showed an increase in VEGF which was found to be positively correlated with ΔCD133^+^/KDR^+^ EPCs (*r* = 0.67, *P* = 0.0045) but not with ΔCD34^+^/KDR^+^ EPCs (*r* = 0.045, *P* = 0.86) (Mobius-Winkler et al. 2009). Moreover, Bonsignore et al. (2010) did not find an increase in VEGF-A but only in VEGF-C which is known to play a role in lymphagiogenesis (Oh et al. 1997). Possible differences between the two trials can be accounted to the different modality used (cycling vs running). It has been shown that exercising at the same intensity, plasma lactate levels, heart rate and carbohydrate oxidation rates are higher during cycling compared to running possibly due to the more localised muscle stress (Capostagno and Bosch 2010). Mobius-Winkler et al. (2009) found that VEGF significantly peaked at the first 10 min during cycling. The increase in VEGF possibly preceded the increase of EPCs in the circulation and it is not known if the same pattern exists during a marathon race (running). Another angiogenic factor found to be increased after prolonged endurance exercise was SCF (Mobius-Winkler et al. 2009). SCF is a hypoxia-inducible cytokine that enhances neovascularisation of EPCs (Kim et al. 2011). However, limited research exists regarding the effects of exercise on circulating SCF and the relationship with EPCs.

Finally, circulating endothelial cells (CD146^+^) which are an indicator of endothelial injury (Blann et al. 2005), were found to be significantly elevated during and following prolonged strenuous cycling (Mobius-Winkler et al. 2009). This possibly suggests that prolonged strenuous exercise induces an increased shear stress that causes a non-permanent endothelial disturbance and EPCs in turn assist in maintaining endothelial integrity.

To conclude further research is required to elucidate the mechanisms by which prolonged strenuous exercise relates to changes in the population of EPCs, while an increase in inflammatory markers such as IL-6 and prolonged disturbance of the endothelium may play some role to exercise-induced EPC mobilisation. The role of angiogenic factors such as VEGF and SCF warrant further investigation.

In the present systematic review, we provide strong evidence that a single RE session can increase circulating EPC numbers (Ross et al. 2014; Kruger et al. 2015; Ribeiro et al. 2017; Montgomery et al. 2019). These changes have been found to occur immediately post-exercise (Ribeiro et al. 2017; Montgomery et al. 2019) and last up to 24 h post-exercise (Kruger et al. 2015; Ribeiro et al. 2017). The effects of an acute RE bout on circulating EPCs is consistent in both young females (Ribeiro et al. 2017) and young males (Ross et al. 2014; Kruger et al. 2015; Montgomery et al. 2019). Moreover, the type and the structure of the RE protocol seem to influence the degree of the increase of circulating EPCs. A high intensity whole-body RE performed at three different intensities (80%1RM, 70%1RM and 60%1RM) and involving four exercises (3 sets × 12 repetitions with 1 min recovery between sets) resulted in more pronounced increases in circulating EPCs (81.1% at 80%1RM) while increases at lower intensities were more modest (43.1% at 70%1RM and 24.3% at 60%1RM) (Ribeiro et al. 2017). Exercising at 80% and 70% likely resulted in the higher acute metabolic responses and disturbance in acid/base homeostasis potentially explaining the different responses. We indeed have previously shown in our laboratory that performing RE between 70–85% of 1RM results in significant metabolic disturbances (Nicholson et al. 2014, 2016). However, neither blood lactate nor blood gases were reported in the latter study. Disturbance in acid–base homeostasis during RE appears to be a causative factor, which is worth exploring further, for an increase in EPC levels. Preliminary findings by Ross et al. (2014) indeed demonstrate that a whole-body RE protocol resulted in significant elevations in blood lactate (11.9 ± 0.9 mmol.L^−1^) alongside an increase in EPC levels by $\sim$ 51.8%. It is also worth noting that the larger increases were observed in two distinct RE protocols. Montgomery et al. (2019) found an $\sim$ 142.6% increase on CD34^+^/KDR^+^/CD45^dim^ EPCs using a unilateral low intensity knee extension (20%1RM), while Kruger et al. (2015) found the most pronounced increases by $\sim$ 405% using a whole-body RE protocol (75%1RM) that lasted 90 ± 5 min. Finally, the latter protocol showed the largest increase compared to an eccentric treadmill running and high intensity concentric cycling protocols.

Several pro-angiogenic factors were examined in RE-based trials to elucidate the mechanisms of EPC mobilisation. There is strong evidence that circulating levels of VEGF increase after a RE bout and their kinetics seem to be mode (Ribeiro et al. 2013; Ross et al. 2014) and intensity dependant (Ribeiro et al. 2017). Moreover, the changes in VEGF were positively correlated with EPC changes 6 h post-exercise (*r* = 0.492, *P* = 0.002) (Ribeiro et al. 2017). Under hypoxic conditions VEGF is upregulated by HIF-1α (Forsythe et al. 1996). Ribeiro et al. (2017) found that after a RE session (both at 70%1RM and 80%1RM) there was an increase in plasma HIF-1α. These increases were positively correlated with EPC changes (*r* = 0.388, *P* = 0.016). From all the above, it can be postulated that the local muscle fatigue (increased blood lactate levels) induced by RE caused an ischaemic stimulus that stimulated HIF-1α which in turn upregulated VEGF and then stimulate EPC mobilisation into circulation.

Erythropoietin is a glycoprotein with pro-angiogenic characteristics which has been shown to exert similar effects with VEGF on EPC mobilisation (Heeschen et al. 2003). Ribeiro et al. (2017) showed that systemic levels of EPO increased post-exercise in all the three RE protocols (but with longer lasting effects only seen with 70% and 80% 1RM). Another pro-angiogenic factor that was found to increase in parallel with circulating EPCs after a RE session was G-CSF (Kruger et al. 2015; Ross et al. 2014). It is well known that G-CSF triggers progenitor cell mobilisation by the release of elastase from neutrophils and MMPs (Greenbaum and Link 2011). Indeed, Ross et al. (2014) found an increase in MMP-9 after the RE session. The critical role of MMP-9 has been demonstrated in MMP-9^−/−^ (MMP-9 deficient) mice where the EPC (Sca-1^+^/FIk-1^+^) mobilisation was blunted after hind-limb ischaemia and VEGF administration whereas in MMP-9^+/+^ mice it was not (Huang et al. 2009). To conclude, the underlying mechanism of RE-induced EPC mobilisation seems to involve local muscular fatigue and ischaemia that act as potent triggers to HIF-1α, VEGF, EPO,G-CSF and MMP-9 where synergistically enhance EPC mobilisation into circulation. However, there is a need for further research to investigate the interactions of several pro-angiogenic factors and their kinetics after exercise with circulating EPCs to elucidate their potential role to EPC mobilisation.

Strong evidence exists that an acute bout of maximal exercise can increase the number of circulating EPCs (Bonsignore et al. 2010; Shill et al. 2016; Van Craenenbroeck et al. 2008b; Yang et al. 2007). Moreover, it has been found that the most commonly used exercise form is a maximal stress test on either a treadmill or a cycle ergometer, with only one trial using 1500 m field test (Bonsignore et al. 2010). In addition, despite the fact that all maximal exercise test trials used the same EPC phenotype (CD34^+^/KDR^+^), one trial failed to find a change post-exercise in any of the groups assessed (Thijssen et al. 2006). This could be attributed to the methodological differences between several flow cytometry protocols in quantifying CD34^+^/KDR^+^ EPCs (Van Craenenbroeck et al. 2008a). Finally, even though most of the trials recruited male individuals, Shill et al. (2016) showed that a maximal exercise test increased circulating EPCs in women as well. However, the lack of consideration of the menstrual cycle in this study warrants further investigation, given that there is a two-fold increase in CD34^+^/KDR^+^ EPCs during the ovulatory phase (Fadini et al. 2008b).

The underlying mechanisms of exercise-induced EPC mobilisation after maximal exercise is not clearly understood. One of the underlying mechanisms of the maximal exercise-induced EPC mobilisation may involve an increase in NOx availability which has been found to be positively correlated with EPC increase (*r* = 0.70, *P* < 0.05) (Yang et al. 2007). A maximal exercise bout increases blood flow and shear stress (Tanaka et al. 2006), which consequently increases the production of endothelial nitric oxide synthase derived NOx, which in turn upregulates EPC mobilisation (Aicher et al. 2003). Contrastingly, Van Craenenbroeck et al. (Van Craenenbroeck et al. 2008b) failed to find an increase in NOx metabolites despite an increase in circulating EPCs. The authors explained findings due to the methodological challenges of analysing NOx. Other pro-angiogenic factors analysed were VEGF and SCF which only the latter found to be significantly increased after maximal exercise (Bonsignore et al. 2010). It has been suggested that during maximal exercise a shift towards glycolytic metabolism is associated with an increase in oxidative stress which in turn creates a hypoxic stimulus, conducive for EPC mobilisation (Van Craenenbroeck et al. 2008b). However, the lack of increase in VEGF may be attributed to the absence of maximal exercise-induced ischaemia in healthy individuals (Thijssen et al. 2006; Yang et al. 2007) which is necessary for VEGF activation (Tilling et al. 2009). More research is warranted to elucidate the precise mechanisms of EPC mobilisation after maximal exercise with some evidence indicating that increased shear stress and NOx production may at least in part play a role.

The most studied form of exercise for EPC mobilisation after acute exercise is a MICON exercise protocol. However, not all trials found an increase post-exercise. This discrepancy between the trials can be attributed to several reasons such as the use of different EPC phenotypes (Niemiro et al. 2017) and the use of small sample sizes to detect significant changes in such a rare cell population (Stromberg et al. 2017). Moreover, there was a large variability of the post-exercise time points associated with significant increases in EPCs immediately post-exercise (Ross et al. 2018), 20 min post-exercise (Cubbon et al. 2010) and 24 h post-exercise (Chang et al. 2015). Niemiro et al. (2017) was the only trial that examined the kinetics of CD45^−^/CD34^+^/CD31^+^ EPCs but failed to find any changes during or up to 120 min post-exercise. Finally, from the data it is not totally clear if intensity or duration play more important role during MICON exercise because of the large variability of exercise prescription and research design. However, a trial that examined this found that the duration, rather that the intensity during MICON exercise seems to be more important for EPC mobilisation (Laufs et al. 2005). Some trials utilising a MICON protocol assessed also the influence of ageing (Lockard et al. 2010; Ross et al. 2018), sex (Adams et al. 2013; Lansford et al. 2016), ethnicity (Cubbon et al. 2010) and fitness status (Lockard et al. 2010).

Regarding the effect of ageing contrasting results exist because one trial showed an increase in CD34^+^/KDR^+^/CD45^dim^ EPCs in the older group (not different to the increase observed in the younger group) (Ross et al. 2018) whereas, the other failed to find any changes in EPCs irrespective of the fitness status (Lockard et al. 2010). Disparity of those findings can be attributed to methodological differences in EPC enumeration between the two trials (e.g., red cell lysed whole blood versus density gradient centrifugation) and different post-exercise blood collection time points (e.g., immediately post-exercise vs 30 min post-exercise).

A MICON exercise protocol does not appear to induce any changes in young female individuals (Lansford et al. 2016). Previously. It has been reported that a maximal exercise test on a cycle ergometer upregulated EPC levels in young women (Shill et al. 2016). Given that both trials used the same EPC phenotype, and had similar baseline characteristics (age, VO_2max_, contraceptive use) it may indicate that exercise intensity plays a role in the exercise-induced EPC mobilisation in women.

Finally, Cubbon et al. (2010) found that United Kingdom based South Asian young individuals displayed a reduced EPC mobilisation to a MICON exercise protocol compared to age matched white Europeans. This reduced response was accompanied by a reduced brachial FMD and increased endothelial microparticles. The latter are known as a surrogate marker of endothelial dysfunction and are associated with the presence of cardiometabolic risk factors (Amabile et al. 2014; Yong et al. 2013). It is noteworthy that the South Asian group had an average VO_2peak_ which was 10 ml.kg^−1^.min^−1^ lower than the White European group’s (Cubbon et al. 2010). Given that circulating EPC levels differ between ethnicities (Murphy et al. 2007; Samman Tahhan et al. 2018), more research is required to identify the optimum exercise mode, intensity and duration based on ethnicity characteristics for circulating EPC mobilisation.

The mechanisms that could explain EPC mobilisation after MICON exercise are hard to delineate based on available evidence, and this is partly due to the large variability of exercise durations and intensities employed across different trials, which in turn elicit different physiological responses (e.g., shear stress, hypoxia). The trials reporting increases in circulating EPCs either did not observe increases in serum VEGF (Laufs et al. 2005) or the increase in plasma VEGF did not correlate with the increases in EPCs (Ross et al. 2018). From that it seems that ischaemia is an unlikely causal factor in EPC mobilisation after MICON exercise in healthy individuals (Laufs et al. 2005). Other circulating chemoattractants such as G-CSF were found to be unaltered after a MICON exercise bout (Stromberg et al. 2017; Ross et al. 2018) whereas, SDF-1α responses seem to be dependent on exercise modality. An exercise bout on a cycling ergometer did not alter circulating SDF-1α (Ross et al. 2018; Stromberg et al. 2017) whereas, a MICON exercise bout on a treadmill increased SDF-1α levels during (Niemiro et al. 2017) and following exercise (Chang et al. 2015; Niemiro et al. 2017). Because SDF-1α and its receptor chemokine receptor 4 play an important role in progenitor cells homing and trafficking (Wen et al. 2012) more research is required to identify the effects of different exercise modalities (cycling vs running) on SDF-1α and consequently EPC mobilisation.

Finally, the main plausible mechanism for MICON exercise-induced EPC mobilisation is the increased NOx availability caused by the increased shear stress in the vascular walls. This was demonstrated by Cubbon et al. (2010) who showed that after the infusion of L-NMMA, a NOx synthase inhibitor the mobilisation of both CD133^+^/CD34^+^/KDR^+^ and CD34^+^/KDR^+^ EPCs following a MICON exercise protocol was essentially absent.

HIIT is a discontinuous mode of exercise which is characterised by short bouts of high intensity exercise, interspersed by periods of rest or low intensity exercise (Meyer et al. 2013; Tschakert and Hofmann 2013). It is reported that long-stage HIIT acutely resulted in higher haemodynamic responses compared to a MICON protocol (Falz et al. 2019). This increased haemodynamic response increased blood flow to the working muscles and in turn can increase the shear-stress-induced NOx bioavailability (Wisloff et al. 2007) which could up-regulate the mobilisation of circulating EPCs (Laufs et al. 2004). However, in the present systematic review the results from the trials that compared HIIT with MICON protocols produced equivocal results. Short-stage HIIT protocols do not have an effect on circulating EPCs both in young (O'Carroll et al. 2019) and post-menopausal women (Harris et al. 2017). Possibly, the exercise stimulus due to the short duration of each exercise bout (10-30 s) was not sufficient to elicit changes in circulating EPCs. In the trials that utilised a long-stage HIIT protocol the results were inconsistent, with one trial reporting decreases in both HIIT and MICON protocols (Sapp et al. 2019), whilst the other reported significant increases without any differences between HIIT and MICON (Kruger et al. 2016). Sapp et al. (Sapp et al. 2019) argued that the decreases in circulating EPCs were due to their recruitment to the blood vessels following their mobilisation. Moreover, it is difficult to compare the two trials because of the different phenotype used to identify EPCs. Further research is required using widely acceptable EPC phenotype and methodology to elucidate the effects of HIIT and if a superiority over MICON exists.

### Chronic effects of exercise on EPCs

In the present systematic review, we provide clear evidence that chronic exercise has positive effects on circulating EPCs. Most of the chronic trials examined the effects of a MICON exercise programme in older individuals. Ageing is a primary risk factor for cardiovascular diseases and it has been associated with endothelial dysfunction (Black et al. 2009), altered microcirculation (Gates et al. 2009), increased oxidative stress (Puca et al. 2013) and decreased NOx bioavailability (Nyberg et al. 2012). Moreover, it is well established that ageing negatively affects the number of circulating EPCs (Thijssen et al. 2006; Yang et al. 2013; Ross et al. 2018). Despite the apparent lack of significant increases in EPC mobilisation in older individuals after an acute bout of exercise in at least one trial (Lockard et al. 2010), the cumulative effects of a MICON exercise programme can lead to increases in circulating EPCs in older men and women (Jo et al. 2019; Landers-Ramos et al. 2016; Xia et al. 2012; Yang et al. 2013) which are paralleled by improvements in FMD (Jo et al. 2019; Landers-Ramos et al. 2016; Xia et al. 2012) and reductions in arterial stiffness (Yang et al. 2013). The beneficial effects of regular exercise training on ageing populations may be attributed to the reduction in oxidative stress, improvement in plasma glucose and NOx bioavailability all known to affect number and function of EPCs (Ross et al. 2016; Williamson et al. 2012), but also to the improvement of intracellular signalling of CXCR-4/JAK-2 which is associated with increased in vitro EPC migratory capacity and adhesion (Xia et al. 2012). The importance of regular physical activity in older populations can be highlighted by the fact that even ten days of detraining were sufficient to reduce the resting levels of CD34^+^/KDR^+^ EPCs in previously trained older men (Witkowski et al. 2010).

In terms of training characteristics, it has been shown that even a short duration (10 day), but intense (daily) MICON exercise programme can increase circulating EPCs in sedentary old individuals (Landers-Ramos et al. 2016). However, one trial surprisingly found a significant decrease in circulating EPCs after 8 weeks of training (Thijssen et al. 2006). When compared to other trials which reported significant increases in circulating EPCs (Xia et al. 2012; Yang et al. 2013), the aforementioned trial (Thijssen et al. 2006) has a reduced total volume of training (8 h vs 18 h) which potentially led to an insufficient exercise stimulus to increase EPC levels. Another factor that can possibly explain the results is the training intensity; however, it is difficult to draw definite conclusions due to the fact that the intensity in some trials which found significant increases in circulating EPCs was either not reported (Xia et al. 2012) or was not reported in detail (Yang et al. 2013). Nevertheless, from the above it can be concluded that most of the available research shows that a MICON exercise protocol has positive effects on sedentary older individuals by increasing circulating EPCs, where those positive effects at least partly can be explained by the training volume.

In the present systematic review, MICON exercise programme produced equivocal findings in healthy overweight/obese individuals. Obesity is a cardiovascular risk factor that has been negatively associated with several circulating EPC subtypes and positively with intima media thickness (Muller-Ehmsen et al. 2008; Sowers 2003). The discrepancy between the trials can be attributed in differences in methodology and design. First, the population examined between the two trials differ in age with Cesari et al. (2012) who found positive effects on all EPC phenotypes having a median age of 43yrs, while Niemiro et al. (2018) with a mean age of 29 ± 8yrs. Previously it has been shown that a 12 week MICON exercise programme produced larger increases in circuiting EPCs in sedentary old adults ($\sim$ 83%) compared to their young counterparts ($\sim$ 22%) (Landers-Ramos et al. 2016). Possibly the need to restore the injured endothelium is greater in elderly individuals compared to young. Also, looking at the training characteristics, in the trial (Cesari et al. 2012) that did find positive effects on circulating EPCs the exercise intervention lasted twice as long (12 weeks vs 6 weeks) compared to the other trial (Niemiro et al. 2018). Therefore, a threshold regarding the length of the exercise programmes possibly could exist for the circulating EPCs levels to rise. Moreover, Cesari et al. (2012) had a large group of participants (n = 40) but analysed them as two separate groups (based on condition). They showed that those who complied to the exercise intervention (completion of all training sessions or > 10% VO_2peak_ improvement) also increased circulating EPCs and those who did not comply showed no increases. It would be useful to know what happened to circulating EPC numbers as a whole group since all participants were recruited as one intervention group without any controls. In contrast, Niemiro et al. (2018) included a small group (n = 10) of obese participants analysed as one group irrespective of their compliance. Their control group (n = 17) consisted of lean individuals who did not have any significant changes in circulating EPCs either. Finally, the two trials differ on the EPC phenotype used to identify this population and at least in one degree can explain the differences in the results.

The mechanisms by which chronic MICON exercise alters circulating EPCs in healthy overweight/obese individuals is poorly understood. Limited information exists from the existing literature with Cesari et al. (2012) reporting a positive relationship between fat mass loss and CD133^+^/KDR^+^ EPCs (r = 0.50, *P* < 0.04). Previously it was demonstrated that weight loss led to increases in plasma adiponectin which was associated with improvements in the lipid profile (Baratta et al. 2004). Circulating adiponectin levels have been positively correlated with circulating EPCs and a reduction of both has been associated with cerebral atherosclerosis and large artery atherosclerotic stroke (Zhang et al. 2016). Also, the effect of adiponectin on EPCs is evident by the protective role of adiponectin on EPC apoptosis by serum deprivation (Lavoie et al. 2011) and an increase in proliferative and functional capacity in a hind-limb ischaemia model (Shibata et al. 2008). From the above it can be speculated that weight loss leads to an increase in adiponectin levels which in turn positively affects the circulating EPCs. However, further research is required to examine the effects of exercise on circulating EPCs in overweight/obese individuals and elucidate the relationship with circulating levels of adiponectin.

Despite the limited literature that exists regarding the chronic effects of HIIT on circulating EPCs and the comparison with MICON exercise, this systematic review showed that short-stage HIIT (10 s or 30 s intervals) is not sufficient to increase circulating EPCs and FMD (Rakobowchuk et al. 2012). In contrast, long-stage HIIT (4 min intervals) was able not only to increase circulating EPCs but elicited increases in circulating EPCs of monocytic origin and led to an improvement in circulating angiogenic cell (CAC) migratory capacity and tube formation (Tsai et al. 2016).

Mechanistically, long-stage HIIT seems to allow more time for repetitive shear stress in the vascular walls compared to short-stage HIIT. A previous meta-analysis found that long-stage HIIT resulted in greater improvements in FMD compared to short-stage HIIT (Ramos et al. 2015). An insight into the possible superiority of HIIT over MICON comes from the study by Tsai et al. (2016) who found that although both exercise modalities induced significant elevations in NOx, HIIT elicited greater improvements. Also, pro-angiogenic factors such as VEGF, MMP-9 and SDF-1a only increased after HIIT. Therefore, the proposed mechanism of HIIT-induced EPC mobilisation is that the increased shear stress activates enzymes such as endothelial nitric oxide synthase in the presence of VEGF. VEGF is known to activate the PI3-kinase/Akt (PI3k/Akt) pathway which stimulates NOx production. The increased NOx levels lead to an upregulation of MMP-9 which increases EPCs (Huang et al. 2009). Also, the increase of MMP-9 causes the release of soluble kit ligand which is essential for the efflux of EPCs into the circulation (Heissig et al. 2002). When EPCs are liberated into the circulation chemokines such as SDF-1α and its receptor chemokine receptor 4 assist them to home into the vascular wall (Schuler et al. 2013).

### Strengths and limitations

To our knowledge, this is the first systematic review examining both the acute and chronic effects of different exercise modalities on circulating EPCs assessed solely by flow cytometry in healthy populations. Previous systematic reviews (Silva et al. 2012) and a meta-analysis (Schmid et al. 2021) examined the acute effects of exercise on EPCs in healthy populations. However, in their searching strategy and analysis they also included trials that analysed CFU-ECs. This assay first introduced by Hill et al. (2003) and found that CFU-ECs were correlated with Framingham risk score and FMD. However, these cells are a heterogenous population with a haematopoietic origin, did not correlate with CD34^+^/KDR^+^ EPCs analysed by flow cytometry and cannot be a reliable assay to enumerate EPCs in peripheral blood (Shantsila et al. 2007; Van Craenenbroeck et al. 2008a; George et al. 2006). While flow cytometry is considered the gold standard for valid and reliable quantification of circulating EPCs, up to date there is no universally agreed EPC phenotype (Fadini et al. 2012; Van Craenenbroeck et al. 2013). In the present systematic review, we identified 8 different EPC phenotypes in acute trials and 5 phenotypes in chronic trials. In addition, it has previously been demonstrated that there is a poor to moderate agreement between 6 different flow cytometric methods (Van Craenenbroeck et al. 2008a) suggesting that one of the biggest hurdles in the present research area is the standardisation of the flow cytometric analysis of circulating EPCs.

From the acute trials it was found that prolonged exercise (> 3 h) and maximal exercise (VO_2max_ test) were two modalities that elicited significant increases in circulating EPCs. However, both cannot be applied in any real-life exercise programme. Given that long-stage HIIT and RE regimes also showed favourable effects on circulating EPCs, future research should aim to compare several real-life applicable exercise modalities and investigate if there is any superiority among them. Concurrent analysis of several pro-angiogenic and inflammatory markers associated with EPC mobilisation can shed a light on the probable mechanisms of exercise-induced mobilisation for each different exercise modality.

In chronic trials no evidence exists yet from interventions utilising a RE protocol. Given that the majority of the trials showed that chronic MICON exercise interventions can increase circulating EPCs in healthy elderly individuals and that RE acutely increases EPC numbers, future research should examine both the acute and chronic effects of RE protocols in the older population.

The results from this systematic review included predominantly males both for acute (74%) and chronic trials (62.4%). Very few trials included solely a female population (Harris et al. 2017; Jo et al. 2019; Lansford et al. 2016; Ribeiro et al. 2017; Shill et al. 2016). Future research should examine the acute and chronic effects of long-stage HIIT and chronic effects of RE in female populations.

### Conclusions

Despite the variations in EPC phenotypes and flow cytometry protocols employed by the included trials, this systematic review provides a comprehensive analysis of the current literature for the exercise modalities employed to investigate the exercise-induced (chronic and acute) effects on EPC mobilisation.

In the acute trials prolonged endurance and resistance exercise have a more lasting effect on EPC mobilisation followed by maximal exercise. Long-stage HIIT seems more favourable for inducing changes in circulating EPCs compared to short-stage HIIT. Some evidence for favourable effects on EPCs from concentric and eccentric endurance exercise exists while MICON exercise was the regime that produced the most equivocal results.

In the chronic trials, evidence suggests that MICON exercise increases circulating EPCs in sedentary older individuals, and this is also paralleled by improvements in endothelial function and reduced arterial stiffness. Similar to acute trials a short-stage HIIT does not appear to increase circulating EPCs, whereas long-stage HIIT not only increased circulating EPCs but overall seem to be superior to a MICON exercise protocol.

**Fig. 2:**
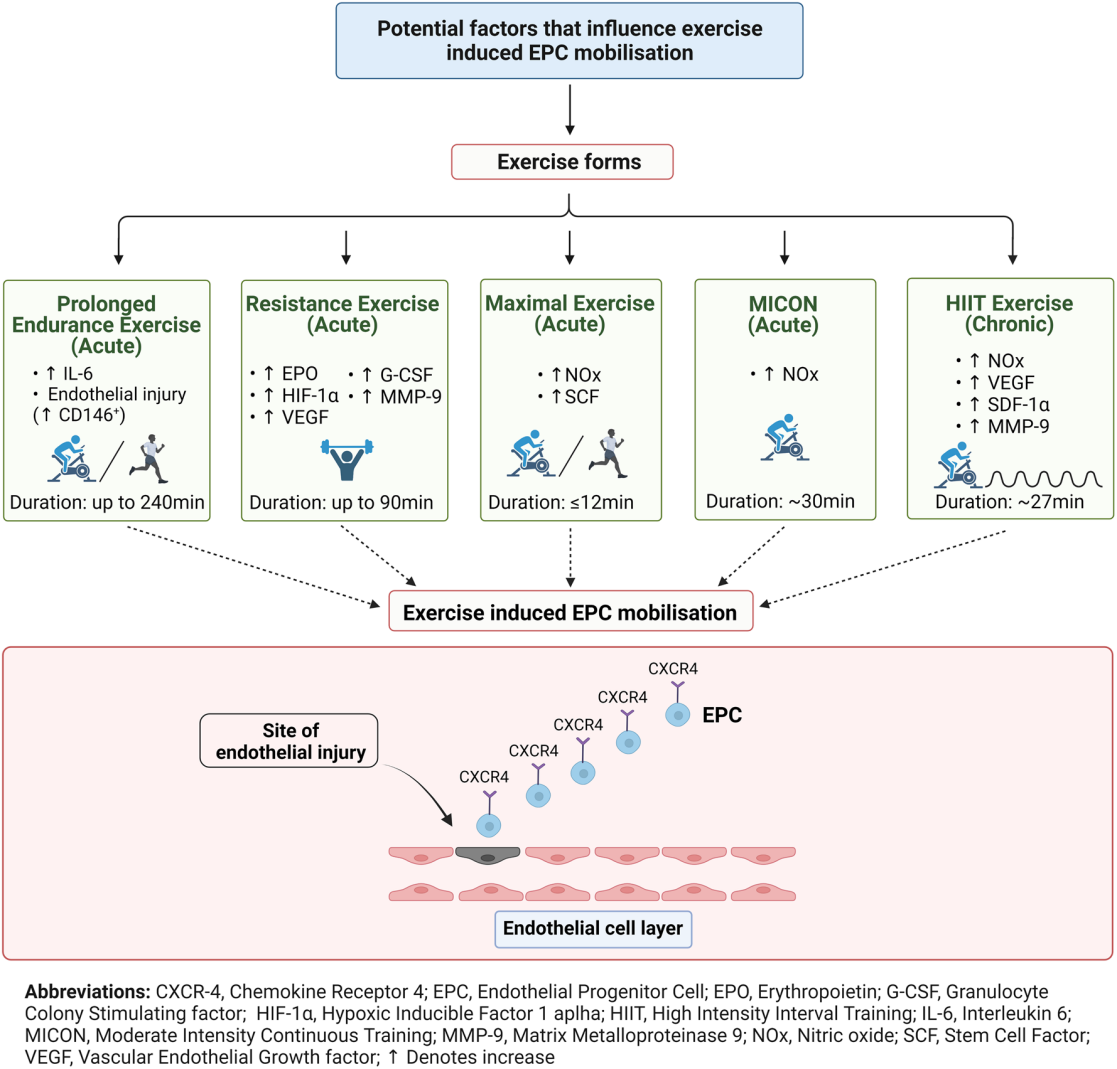
A schematic diagram summarising current evidence regarding potential regulatory factors of endothelial progenitor cell mobilisation and “homing” to the site of injury, for different exercise modalities. Exercise induced increase in shear stress activates the endothelial nitric oxide synthase (eNOS), which in turn stimulates NOx bioavailability. Several angiogenic factors such as VEGF, MMP-9, G-CSF and EPO, assist the mobilisation of EPCs into the circulation. After released into the circulation, EPCs are attracted to the site of the endothelial injury by CXCR-4 (receptor for SDF-1α) and consequently involved to the repair of the damaged endothelium. Other potential factors for EPC mobilisation and recruitment are endothelial damage (increased CD146 + cells), increased inflammation (IL-6) and muscle ischaemia (increased HIF-1α and SCF)

The mechanisms of exercise-induced EPC mobilisation are dependent on the exercise mode, with NOx availability, inflammation (IL-6), muscle fatigue and increase of growth factors (VEGF) and chemokines (SDF-1a) all appearing to play a role in the upregulation of the release and mobilisation of EPCs (Fig. 2).

In the future, a variety of real-life exercise regimes should be employed to explore the optimum intensity, duration, and frequency for the optimisation of EPC mobilisation using universally accepted EPC phenotypes and flow cytometry methodologies. Finally, the large range of pro-angiogenic factors should be examined along with the circulating EPCs to fully understand the mechanisms of exercise-induced EPC mobilisation.

## Author contributions 

PF, CT, MS, MMS, AD and TI: Conceptualization. PF, CT, MS, MMS, AD and TI: Methodology. PF, CT, MS: Investigation and data curation. PF, CT, and TI: Formal analysis. PF, CT, and TI: Writing original draft. PF, CT, MS, MMS, AD and TI: Writing-Reviewing and Editing

## Supplementary Information

Below is the link to the electronic supplementary material.

Supplementary file1 (DOCX 13 KB)

Supplementary file2 (DOCX 18 KB)

## Abbreviations

1RM: One repetition maximum strength
EPCs: Endothelial progenitor cells
FMD: Flow-mediated dilatation
G-CSF: Granulocyte colony stimulating factor
HIF-1α: Hypoxia-inducible transcription factor 1 alpha
HIIT: High intensity interval training
IL-6: Interleukin 6
MICON: Moderate intensity continuous training
MMP-9: Matrix metalloproteinase 9
NOx: Nitric oxide metabolites (Nitrite/Nitrate)
PBMNCs: Peripheral blood mononuclear cells
SCF: Stem cell factor
PRISMA: Preferred reporting items for systematic reviews and meta-analyses
RE: Resistance exercise
SDF-1α: Stromal cell derived factor 1 alpha
SWiM: Synthesis without meta-analysis
TESTEX: Tool for the assessment of study quality and reporting in exercise
VEGF: Vascular endothelial growth factor
VO_2peak/max_: Peak/maximal oxygen uptake
